# Cognition and Activity of Daily Living Function in people with Parkinson’s disease

**DOI:** 10.1007/s00702-024-02796-w

**Published:** 2024-07-08

**Authors:** Merle Bode, Elke Kalbe, Inga Liepelt-Scarfone

**Affiliations:** 1 Hertie Institute for Clinical Brain Research, Department of Neurodegenerative Diseases, Eberhard Karls University Tübingen, Hoppe-Seyler Str. 3, 72076 Tübingen, Germany; 2 German Center for Neurodegenerative Diseases (DZNE), Tübingen, Germany; 3 Medical Psychology | Neuropsychology and Gender Studies & Center for Neuropsychological Diagnostics and Intervention (CeNDI), University Hospital Cologne, Cologne, Germany; 4 Medical Faculty, University of Cologne, Cologne, Germany; 5 IB-Hochschule, Stuttgart, Germany

**Keywords:** Parkinson’s disease, Activities of daily living, Cognition, Neuropsychology, Physical activity, Sedentary behavior

## Abstract

The ability to perform activities of daily living (ADL) function is a multifaceted construct that reflects functionality in different daily life situations. The loss of ADL function due to cognitive impairment is the core feature for the diagnosis of Parkinson’s disease dementia (PDD). In contrast to Alzheimer’s disease, ADL impairment in PD can be compromised by various factors, including motor and non-motor aspects. This narrative review summarizes the current state of knowledge on the association of cognition and ADL function in people with PD and introduces the concept of “cognitive ADL” impairment for those problems in everyday life that are associated with cognitive deterioration as their primary cause. Assessment of cognitive ADL impairment is challenging because self-ratings, informant-ratings, and performance-based assessments seldomly differentiate between “cognitive” and “motor” aspects of ADL. ADL function in PD is related to multiple cognitive domains, with attention, executive function, and memory being particularly relevant. Cognitive ADL impairment is characterized by behavioral anomalies such as trial-and-error behavior or task step omissions, and is associated with lower engagement in everyday behaviors, as suggested by physical activity levels and prolonged sedentary behavior. First evidence shows that physical and multi-domain interventions may improve ADL function, in general, but the evidence is confounded by motor aspects. Large multicenter randomized controlled trials with cognitive ADL function as primary outcome are needed to investigate which pharmacological and non-pharmacological interventions can effectively prevent or delay deterioration of cognitive ADL function, and ultimately the progression and conversion to PDD.

**Supplementary Information:**

The online version contains supplementary material available at 10.1007/s00702-024-02796-w.

## Introduction

In this narrative review, we present the current state of knowledge on the association between cognition and activities of daily living (ADL) function in Parkinson’s disease (PD) and introduce the term *cognitive ADL* *impairment* for those problems in everyday life associated with cognitive deterioration.

First, we provide a brief overview of cognitive impairment in PD. Cognitive impairment is one of the most common and troublesome non-motor symptoms in people with PD. The occurrence and severity of cognitive impairment in PD is associated with shorter life expectancy, contributes to distress amongst caregivers, and increases the risk of nursing home placement (Bjornestad et al. 2017). Approximately 60% to 83% of people with PD develop Parkinson’s disease dementia (PDD) during the course of their disease (Hely et al. 2008), and about 20 to 33% of patients experience mild cognitive impairment (PD-MCI) already at the time of PD diagnosis (Lawson et al. 2017; Santangelo et al. 2015). Cognitive functions, particularly executive functions such as verbal fluency performance (Darweesh et al. 2017b; Behnke et al. 2007), and set shifting (Thaler et al. 2012) may already be impaired in the prodromal stage of PD. Moreover, working memory performance (Darweesh et al. 2017a) and memory complaints (Foubert-Samier et al. 2020; Schrag et al. 2015) were found to be associated with PD conversion. Additionally, reduced visual memory performance has also been reported in PD-free glucocerebrosidase (GBA) mutation carriers (Zokaei et al. 2014) and impaired verbal memory performance (Yilmaz et al. 2016; Holthoff et al. 1994; Chahine et al. 2015) in PD risk groups. These results emphasize that cognition may decline even before the PD motor symptoms are severe enough to justify the clinical diagnosis of PD (Fengler et al. 2017; Liepelt-Scarfone et al. 2022; Berg et al. 2012). The profile of cognitive impairment in PD is heterogeneous (Aarsland et al. 2021; Kehagia et al. 2010). Executive dysfunction, such as reduced information generation, response inhibition, rule deduction, dual-task performance, or set-shifting occur early in the disease stage and are modulated by frontal-striatal alterations (Dirnberger and Jahanshahi 2013). Episodic memory retrieval and metacognitive processes are also affected by executive control (Dirnberger and Jahanshahi 2013). Furthermore, difficulties in working memory performance and immediate and delayed free recall (Kalbe et al. 2016; Muslimovic et al. 2005; Warden et al. 2016), as well as problems in goal-directed allocation of attention, internal attentional control (Bin Yoo et al. 2018), and visuo-cognitive skills (e.g. visuo-constructive abilities with command tasks; Liebermann-Jordanidis et al. 2022) frequently occur in PD. Language deficits, such as naming difficulties or sentence production with reduced information content have been reported in later stages of PD (Hobson and Meara 2004; Small et al. 1997). However, there is increasing evidence that language deficits occur in all disease stages and that complex sentence comprehension deficits are linked to basal ganglia damage in people with PD (Novakova et al. 2023; Altmann and Troche 2011). These cognitive difficulties may have a detrimental impact on the patents’ ability to function effectively in everyday life. For example, executive functions are regarded as crucial to maintain everyday skills (Godefroy et al. 2010; Koerts et al. 2011) and impaired cortical executive control may account for lost automaticity even in formerly routine tasks (Ferrazzoli et al. 2018; Dirnberger and Jahanshahi 2013).

Preventing or delaying PDD is a matter of great urgency due to its devastating impact on patients and caregivers. Besides older age, the presence of PD-MCI is an established risk factor for PDD (Litvan et al. 2011). PD-MCI can be defined as the intermediate stage of cognitive impairment between normal cognitive function and dementia. Frontal-striatal pathological changes may originate from nigrostriatal and subsequent mesocortical dopamine denervation (Kehagia et al. 2010). Loss of noradrenergic innervation, originating in the locus coeruleus, has also been reported in people with PD-MCI, whereas serotonergic dysfunction is not directly related to cognitive decline (for details see Aarsland et al. 2021). Extensive Lewy body pathology, widespread cortical and subcortical degeneration, and a profound cholinergic deficit may increase the risk for dementia (Kehagia et al. 2013; Williams-Gray et al. 2009). According to the dual-syndrome hypothesis (Kehagia et al. 2010, 2013), cognitive deficits are not limited to executive dysfunction and memory impairments, which are observed in early-stage cognitive impairment in PD, but also include other distinctive cholinergic visuospatial, memory and psychiatric deficits associated with PDD (Aarsland et al. 2021; Kehagia et al. 2010). Of those, worsening of visuo-cognitive skills has been reported to predict cognitive impairment and PDD (Mahieux et al. 1998; Williams-Gray et al. 2009), and language problems occur in the transition to PDD (Hobson and Meara 2004). For an overview of possible neural substrates of specific cognitive functions, we refer to Aarsland et al. (2021) and Citro et al. (2024).

However, PD-MCI is a heterogeneous concept; a recent meta-analysis found that, on average, 31% of PD-MCI patients converted to dementia within seven years; but 24% of PD-MCI patients reverted back to normal cognitive function (Saredakis et al. 2019). This heterogeneity highlights that additional markers are needed to identify those PD-MCI patients that are at high risk for the development of PDD.

Established diagnostic criteria have defined ADL impairment with impact on patients’ personal, occupational, and social daily life due to cognitive deterioration as the core feature differentiating PDD from PD-MCI (Dubois et al. 2007; Emre et al. 2007). It has been reported that the onset of early signs of ADL impairment is a potential risk marker for PDD, which can already be observed in about 30% to 50% of PD-MCI patients (Glonnegger et al. 2016; Martin et al. 2013). People with PD-MCI who display exclusive impairment in ADL tasks that are cognitively demanding and more complex are at an increased risk to develop PDD within three years (Becker et al. 2022a). This finding suggests that the emergence of ADL impairment associated with cognition (cognitive ADL impairment) could serve as an important clinical marker for future PDD, potentially lowering the false-positive rate in people with PD-MCI. In order to pave the path for future research, it is therefore crucial to enhance our understanding of the relationship between cognition and ADL function in PD.

## ADL impairment associated with cognition in PD

### Definition of cognitive ADL impairment and theoretical conceptualization

The evaluation of a patient’s ability to lead an independent life within their community is a key element of clinical practice for the diagnosis of cognitive impairment. Because ADL function can be viewed as a multifaceted construct that reflects functionality in different daily life situations, it is thought to offer a holistic understanding of the patient’s health within the broader context of their physical, emotional, and social well-being (Bezdicek et al. 2023; Bruderer-Hofstetter et al. 2020). ADL function is commonly divided into basic ADL, which involve self-maintenance skills such as bathing, dressing, or eating, and instrumental ADL (IADL), which involve more complex tasks such as medication management, grocery shopping, or financial administration (Lawton and Brody 1969). More severe ADL impairment in PD has been associated with demographic factors (e.g. higher age, male sex), progressive motor impairment, and non-motor symptoms such as depression (Holroyd et al. 2005; Lang et al. 2013; Sperens et al. 2020). The neurobiology of ADL impairment in PD is still unclear. Lower global ADL function has been linked to reduced functional connectivity in people with PD (Yoo et al. 2015). Beta-Amyloid burden has been shown to be related to ADL impairment in non-PD older adults with MCI, although this association has not yet been confirmed in PD (Jutten et al. 2019).

As mentioned before, losing the ability to perform ADL tasks due to cognitive dysfunction is mandatory for the diagnosis of PDD (Dubois et al. 2007). Therefore, it is necessary to gain insight into how a patient applies their cognitive skills to ADL tasks, that is, real-world situations embedded in a context-rich environment. In order to make a valid diagnosis of PDD, it is important to identify the sources of the patient’s ADL impairment, since PD is primarily a movement disorder. Thereby, distinguishing between different sources of their ADL impairment might be challenging, as motor disturbances also negatively affect ADL function in PD (Lee et al. 2014; Skinner et al. 2015; Rosenthal et al. 2010). For instance, tremors may impede the performance of actions related to meal intake, such as cutting food or bringing a spoon to the mouth without spilling the food (Hanff et al. 2022; Westergren et al. 2016). Additionally, falls frequently occur during everyday behaviors that involve sit-to-stand transfers, walking, bending, reaching, or standing (Allen et al. 2013; Ashburn et al. 2008) and evidence indicates that people with PD who are afraid of falling avoid many daily life situations (Bezdicek et al. 2022; Rider et al. 2023), engaging in less bodily behaviors that involve movement (Atrsaei et al. 2021; Landers et al. 2021). These observable motor aspects may influence the patients’ and the caregivers’ ADL ratings specifically in those ADL tasks that involve substantial physical movement (Becker et al. 2020). On the other hand, an ADL task such as making a doctor’s appointment requires the patient to remember their doctor’s phone number and to effectively communicate in order to match available times with their personal calendar, all while sustaining noisy distractions in the background. This ADL task could therefore be regarded as more cognitively demanding and potentially more sensitive to cognitive dysfunction. The comparison between these two ADL tasks illustrates that situations in everyday life may be qualitatively different in terms of their demands on cognitive abilities and motor skills.

However, distinguishing between the sources of ADL impairment may not always be straightforward. For example, dialing the doctor’s phone number might be more difficult with reduced fine motor function (Lopez et al. 2019; Staunton et al. 2022). The level of required movement may also differ between ADL tasks, and hence their susceptibility to motor confounds may differ accordingly. Motor-related difficulties, such as spilling food can also cause stress or shame in patients (Thordardottir et al. 2014). This may lead them to withdraw from public and social activities to avoid these aversive feelings (Ahn et al. 2022; Thordardottir et al. 2014), a behavior that may be similar to the social isolation frequently observed in dementia (Geng et al. 2024; Lazzari and Rabottini 2022; Shen et al. 2022). Depressive symptoms and apathy, regardless of the presence of motor symptoms, can lead to difficulties in coping with everyday life because persons may lack motivation and interest in activities and experience a loss of energy (Laatu et al. 2013; Numbers et al. 2022). The risk of depression is higher in people with PDD than in those without cognitive impairment, i.e., both phenomena often occur together and have an impact on everyday life (Riedel et al. 2010). Thus, a valid differential diagnosis of depression and dementia is of great importance for further treatment decisions. Another non-motor symptom is autonomic dysfunction, bladder disorders in particular, which patients identify as having a substantially negative impact on their everyday life (Allan et al. 2006). To support the diagnosis of PDD, these diverse factors affecting ADL function should be considered in order to identify if cognitive dysfunction is the primary factor of a patient’s ADL impairment. Therefore, understanding which ADL tasks are more appropriate for detecting cognitive ADL impairment is essential to support the diagnosis of PDD.

The findings presented above show that ADL impairment cannot be regarded as a universal construct. It was reported that people with PD-MCI encountered more difficulties with travelling outside of the house, involving motor actions such as walking, going by car or by bus, than people with Alzheimer’s disease MCI (Becker et al. 2021). Difficulties in these actions could hence lead to an overestimation of ADL impairment or even false positive diagnosis of functional dependence (Almeida et al. 2017). Conversely, these findings may also suggest that ADL impairment of a patient with more impaired cognition, but less severe motor symptoms, could be underestimated in an assessment that primarily focuses on motor-demanding ADL. These issues underscore the importance of carefully deconstructing the demands of ADL tasks for an accurate clinical assessment, in order to avoid overestimating or underestimating ADL impairment.

Fortunately, the awareness for these issues is growing in research. Attempts of deconstructing ADL demands show that it is possible to distinguish between ADL tasks based on their reliance on different skills. For example, factor analytic and regression-based approaches have found the ADL task of financial management to be cognitively more demanding (Becker et al. 2020; Sulzer et al. 2020), as compared to the ADL of shopping, which was more influenced by motor symptoms (Becker et al. 2020; Cheon et al. 2015). Furthermore, certain ADL tasks appear to be more affected by depressive symptoms than others (Choi et al. 2019). Most important, novel assessments have been developed (PASS and PDAQ-15; Foster et al. 2014; Holden et al. 2018) to evaluate performance in ADL with greater cognitive demands, and there have also been suggestions for adapting existing clinical measures (Almeida et al. 2017; Becker et al. 2020; Cheon et al. 2015). Based on these issues, we introduce the term cognitive ADL impairment for those ADL tasks that are most likely to be affected by cognitive impairment in PD.

### ADL impairment and cognitive diagnosis in PD

Comparisons among cognitive subgroups in PD show that ADL impairment worsens with cognitive impairment: People with PDD show the most pronounced ADL impairment, specifically in IADL, compared to non-demented people with PD, followed by people with PD-MCI compared to cognitively normal PD (PD-NC) patients (Almeida et al. 2017; Beyle et al. 2018; Cholerton et al. 2020; Giovannetti et al. 2012; Glonnegger et al. 2016; Becker et al. 2020; Cheon et al. 2015; Holden et al. 2018; Fellows and Schmitter-Edgecombe 2019; Kulisevsky et al. 2013).

Therefore, research indicates that cognition is closely intertwined with ADL function, with initial ADL problems being already evident in PD-MCI. Despite these findings, understanding of the progression of ADL function associated with cognitive worsening in PD has been largely neglected (Martin et al. 2013). Additionally, ADL impairment increases within the transition phase of PDD converters (Becker et al. 2022a; Beyle et al. 2018), and presence of cognitive ADL impairment increases the risk for PDD conversion within three years (Becker et al. 2022a). However, not all studies have confirmed the direct link between cognition and ADL function in PD (Reginold et al. 2012; Pirogovsky et al. 2014). Different ways to clinically assess cognitive ADL function will be presented below, followed by their cognitive correlates. Then, an outlook on novel assessments will be provided, and current findings on available interventions will be summarized.

## Evaluating ADL in clinical routine

Assessment of ADL function in PD typically involves self-ratings by the patient or informant-ratings made by a spouse, a close relative, or other knowledgeable informants (Shulman et al. 2016). Thereby, patients and informants may derive their ratings by comparing multiple everyday observations with the patients’ premorbid level of functioning (Schmitter-Edgecombe et al. 2011), hence providing important insights on the personal experiences of within-patients changes. As an alternative to these subjective ratings, performance-based ADL assessments evaluate individuals as they carry out simulated everyday scenarios (Giovannetti et al. 2012; Holden et al. 2018; Sulzer et al. 2020).

Self-ratings of ADL function have not consistently demonstrated the ability to distinguish between cognitive subgroups in PD (Holden et al. 2019; Pirogovsky et al. 2012, 2014). Accurate self-ratings require adequate cognitive functioning, as patients must understand the questions and be aware of their own, even subtle ADL impairments. Accordingly, overestimations of ADL function in people with PD have been associated with decreased awareness (Lehrner et al. 2015), a phenomenon that is observed more frequently in people with cognitive impairment (Pennington et al. 2021; Siciliano et al. 2021) and is at least partly modulated by executive function and attention (Kudlicka et al. 2013; Siciliano et al. 2021). Consequently, there is discordance between self-ratings, informant-ratings, and performance-based ADL assessments, which becomes more pronounced as patients’ cognition decreases (Becker et al. 2020; Deck et al. 2019; Shulman et al. 2006). Self-ratings may primarily be sensitive to ADL impairment in the initial stages of cognitive impairment, as self-ratings of ADL function have been reported to correlate with ADL performance in people with PD-NC but not in those with PD-MCI or PDD (Holden et al. 2019). Importantly, in the initial stages of cognitive impairment, self-ratings may be even more sensitive than informant-ratings because people with PD may be highly alert to any detriment in their ability to perform especially more complex ADL, which may, however, be subtle to informants (Cholerton et al. 2020; Kudlicka et al. 2013). Conversely, informant-ratings may be sensitive to ADL impairment in later stages of cognitive decline, as they demonstrate higher consistency with performance-based ADL assessments than patients’ self-ratings (Becker et al. 2022c; Deck et al. 2019; Shulman et al. 2006). Informant-ratings may, however, be negatively biased due to the informants’ caregiving burden, which has been found to increase with patients’ cognitive impairment (Caap-Ahlgren and Dehlin 2002; Ransmayr 2020). A further issue of rating scales in general is that their accuracy can be reduced by measurement errors. For example, raters may interpret items differently and their responses may be influenced by response tendencies (Matthews et al. 2012; Zolfaghari et al. 2022). Taken together, these findings suggest that cognitive impairment may introduce bias into subjective ADL assessments and that the sensitivity of self-ratings and informant-ratings of cognitive ADL impairment may vary with cognitive deterioration.

Performance-based ADL assessments are directly based on ADL situations, rather than the subjective recall of such, which is the case with subjective ADL ratings (Deck et al. 2019; Schmitter-Edgecombe et al. 2022; Shulman et al. 2006). Comparisons between cognitive subgroups in PD consistently indicate that as cognitive impairment increases, performance-based ADL function also deteriorates (Beyle et al. 2018; Glonnegger et al. 2016; Giovannetti et al. 2012; Holden et al. 2018). In performance-based ADL assessments, individuals are prompted to carry out particular tasks from different ADL domains, such as preparing meals or beverages, shopping, making phone calls, managing medications, using public transportation, or managing finances (Glonnegger et al. 2016; Holden et al. 2018; Sulzer et al. 2020). Most often, ADL function is assessed as overall performance across multiple ADL domains, however, there are also assessments that focus on single ADL domains (Carlson et al. 2005; Marson et al. 2000; Patterson et al. 2002). Thereby, ADL function is evaluated based on quantifiable aspects of the individual’s behavior, such as the number of committed errors, the number of cues provided by the investigators, and the completion time. These aspects are typically protocol documented by external investigators with high inter-rater reliability (Beyle et al. 2018; Giovannetti et al. 2012; Sulzer et al. 2020). Criticism of performance-based ADL assessments may be that they are often time-consuming (Martin et al. 2013; Shulman et al. 2016; Pirogovsky et al. 2013; Roll et al. 2019; Brennan et al. 2016c) and/or involve a high level of material expenditure (Fellows and Schmitter-Edgecombe 2019; Foster 2014). Another shortcoming of performance-based ADL assessments is that they are limited to the laboratory test situation (Warmerdam et al. 2020) and that they may not adequately evaluate intra-individual fluctuations of ADL function as well as the frequency of ADL difficulties in daily life (Cain et al. 2009; Weakley et al. 2019). Moreover, performance may be modulated by several psychological factors that are influenced by the laboratory situation (Paradis and Sutkin 2017; Warmerdam et al. 2020). Despite these limitations, performance-based ADL assessments present an objective and standardized method of assessing cognitive ADL function without cognitive bias outside of an individual’s routines in their home-based environment.

### Clinical ADL assessments to support cognitive diagnosis in PD

The International Parkinson and Movement Disorder Society (MDS) Task Force on Rating Scales recommended the following scales for the assessment of disabilities in PD (see Shulman et al. 2016 for details). Of all scales recommended by the MDS Task Force on Rating scales, only the IADL scale of Lawton & Brody, the UPDRS-II, and the Schwab and England (S&E-ADL) scale have been evaluated in their diagnostic abilities to differentiate cognitive subgroups.

Table 1 gives an overview of various ADL scales which support the diagnosis of PDD, therefore reflecting cognitive ADL impairment. Except for the IADL scale from the Lawton & Brody scale and the Pill-Questionnaire, the diagnostic discriminatory power for PDD of the scales discussed below has only been confirmed in a single study. The IADL scale of Lawton & Brody (Lawton and Brody 1969) is used as a self- and informant-rated assessment (Christ et al. 2013) and includes eight different ADL domains (e.g. shopping, using transport), which are based on items rated on a three-point Likert scale. Group comparisons show that the informant rated scale can differentiate test performance between people with and without PDD (Dujardin et al. 2010; Giovannetti et al. 2012). Scoring of the IADL scale differs, with ratings on a two-point Likert scale (0 = less able or 1 = more able to do the task), resulting in a maximum score of 8, as well as ratings on a four-point Likert score (0 = unable to do the task to 2 = independent), resulting in a maximum score of 24 (Shulman et al. 2016). For both scores, higher values indicate better ADL performance.

**Table 1:** Overview of (instrumental) activity of daily living measures (IADL) with sufficient diagnostic accuracy to support diagnosis of Parkinson’s disease dementia (PDD) vs. non-demented people with Parkinson’s disease (PD)

Measure	Clinometric parameters in PD			Diagnostic accuracy for PDD			
	Reliability	Validity	SE of change	Reference	Cut-off	Sensitivity	Specificity
Self-ratings							
Lawton & Brody IADL	+	+	+	Christ et al. (2013)	< 18	57%	89%
Informant-ratings							
Lawton & Brody IADL	+	+	+				
- max. score 24 points				Christ et al. (2013)	< 19	71%	82%
- max. score 8 points				Tafiadis et al. (2023)	< 7	88%	71%
PDAQ-15	+	+	+	Brennan et al. (2016c)	< 43	82%	84%
PD-CFRS	+	+	+	Kulisevsky et al. (2013)	> 6	83%	83%
Investigator-ratings							
S&E-ADL Scale	+	+	+	Christ et al. (2013)	< 75%	71%	77%
UPDRS-II	+	+	+	Christ et al. (2013)	> 15	67%	78%
Performance-based assessments							
Pill-Questionnaire	NA	+	NA				
				Martinez-Martin (2013)	< 3	92%	91%
				Christ et al. (2013)	< 1	91%	97%
UPSA	+	+	+	Holden et al. (2018)	< 73	80%	89%

*NA* Not available; *PDAQ-15* Penn Parkinson's Daily Activities Questionnaire; *PD-CFRS* Parkinson's Disease Cognitive Functional Rating Scale; PDD Parkinsons’s disease dementia; *S&E-ADL* Schwab and England Activities of Daily Living; *SE* sensitivity; *UPDRS-II* Unified Parkinson's Disease Rating Scale part II; *UPSA* University of California San Diego Performance- Based Skills Assessment

The S&E-ADL Scale (Schwab and England 1968) is rated by physicians, patients, or staff (McRae et al. 2000) using a scale that ranges from 0% to 100% (completely independent; Shulman et al. 2016). The S&E-ADL scale score of people with PDD cannot be differentiated from those with PD-MCI but is able to distinguish PD-NC from people with PD with cognitive impairment (Leroi et al. 2012). The UPDRS is a rating tool designed to allow physicians to monitor worsening or improvement of PD over time in four subparts (Goetz et al. 2008). Its second subpart, the UPDRS-II, covers ADL function, but places greater emphasis on motor limitations than on cognitive limitations. In particular, some of the items of the UPDRS-II enquire about tremor, falling, freezing, or difficulties in swallowing. These items do not represent cognitive ADL impairment and consequently, they can be omitted from the assessment of cognitive ADL function without loss of reliability (Goetz et al. 2008). To date, the discriminatory power of the MDS-UPDRS-II score has not yet been investigated.

In addition to the recommendation of the MDS Task Force on Rating Scales for the assessment of disabilities in PD, good diagnostic discriminatory power of the informant-rated Parkinson’s Disease Cognitive Functional Rating Scale (PD-CFRS) has been suggested for the comparison between people with PDD and non-demented people with PD (Kulisevsky et al. 2013). Studies confirm the reliability and validity of the PD-CFRS, and a change in test score has been reported to correlate with cognitive decline after one year (Kulisevsky et al. 2013; Ruzafa-Valiente et al. 2016; Rosenblum et al. 2022). The performance-based University of California San Diego Performance-Based Skills Assessment (UPSA) was originally designed for the use in schizophrenia (Patterson et al. 2002) and for the assessment of performance in five ADL domains (Financial, Communication, Planning/Organization, Travel, and Household), which are scored separately (range 0–20) and are summed to a total UPSA score (range 0–100). Reliability and validity of the UPSA is good (Holden et al. 2020), but test performance needs to be adjusted for age, education, disease duration, intake of dopaminergic medication, and motor impairment. Sensitivity to change has been recently demonstrated in people with PD (Carlisle et al. 2023). However, baseline scores of the UPSA did not predict the rate of change in cognition over time, and therefore did not identify those individuals at greatest risk of cognitive decline. Discriminatory power of the UPSA for PDD among non-demented people with PD is high, a cut-off score of 83 identified PD-MCI vs. PD-NC with a sensitivity of 79% and a specificity of 64% (Holden et al. 2018).

The Pill Questionnaire has been recommended by a group of PD experts to assess ADL function that is indicative of PDD (Dubois et al. 2007). The test requires people with PD to report intake on their regular Parkinson medication (Dubois et al. 2007). Therefore, the Pill Questionnaire can be considered as a memory task and does not require any demonstration of directly observable ADL functions or transfer performance. Scoring of the scales varies from a graded four-point classification (Reginold et al. 2012; Martinez-Martin 2013; Dubois et al. 2007) of persons’ independence for ADL function and a dichotomized performance rating (Christ et al. 2013). Studies have reported good discriminatory power of the Pill Questionnaire for PDD (Christ et al. 2013; Martinez-Martin 2013). The Pill Questionnaire has a high false-positive rate and potential diagnosis of PDD must be further clarified if the test result is positive (Martinez-Martin 2013). In non-demented people with PD, the Pill Questionnaire has demonstrated low sensitivity (41%) and specificity (80%) in detecting PD-MCI (Reginold et al. 2012). The reliability of the Pill Questionnaire and its sensitivity to change have not yet been proven in PD. In addition, test performance may be negatively influenced by various factors (e.g. presence of apathy, depression, level of education).

Another scale specifically developed to assess cognitive ADL function in PD is the informant-rated Penn Parkinson’s daily activities questionnaire-15 (PDAQ-15, Jonasson et al. 2017). The items of the PDAQ-15 were selected based on experts' opinions from the original 50-item version of the PDAQ (Brennan et al. 2016b), rated on a five-point Likert scale (0 to 4 points). The association between global cognition and the PDAQ-15 has been reported to be large, but the test score was also correlated with the UPDRS-III motor score, though to a minor extent. A cutoff of 37 differentiated PD-MCI from PDD with a sensitivity of 83% and a specificity of 71%. Self-ratings and informant ratings showed moderate agreement on the overall PDAQ-15 (Deck et al. 2019), and self-reported cognitive complaints were predicted by the PDAQ-15 at three years (Weintraub et al. 2024). In addition, the PDAQ-15 demonstrated the ability to reflect treatment-induced improvement of ADL function after deep brain stimulation, indicating good sensitivity to change (Bezdicek et al. 2022). The observer-based Performance Assessment of Self-care Skills (PASS) was also designed to assess cognitively-demanding IADL performance in participants with PD without dementia (Foster and Doty 2021). The PASS score is primarily associated with cognitive function and to a lesser extent with motor performance. Between-group analysis has been shown to differentiate between performance of controls and non-demented PD patients (Foster et al. 2014; Foster and Doty 2021), however, diagnostic accuracy for PD-MCI has not yet been reported.

Almeida et al. (2017) modified the Pfeffer Functional Activities Questionnaire (FAQ, Pfeffer et al. 1982), by including two scale items that, in the authors’ opinion, primarily reflect motor impairments (Almeida et al. 2017). For the modified version of the FAQ, a cut-off of 3.5 points has been proposed based on another validated ADL scale. The cut-off discriminates between PD patients with and without ADL limitations with a sensitivity of 47% and a specificity of 88%. A recent study developed novel ADL scores based on a data-driven approach to discriminate between motor and cognitive contributions to ADL in PD using the FAQ (Becker et al. 2020). People with PD-MCI with more cognitive than motor associated ADL problems had a higher risk of PDD conversion (Becker et al. 2022a), but the discriminatory power of the novel subscores for either PD-MCI or PDD has not yet been defined. The total FAQ score showed a moderate to low sensitivity for PD-MCI (< 55%), but a moderate to high specificity (> 54%), with a tendency of higher values for self-ratings compared to informant-ratings of ADL function (Becker et al. 2022b).

In summary, the evaluation of the potential for ADL measures to detect cognitive ADL impairment is still at its infancy. Although the diagnostic values of various assessments need to be confirmed in independent PD cohorts, there is evidence that ADL assessments have the potential to differentiate cognitive subgroups of people with PD. Of those, the PDAQ-15, the PD-CFRS, and the UPSA show sufficient diagnostic properties and accuracy for PDD and are therefore valuable ADL assessment to support cognitive diagnosis.

### Cognitive correlates of ADL impairment

In cognitively mixed PD cohorts, including different cognitive PD subgroups (e.g. PD-NC, PD-MCI, and PDD; Litvan et al. 2012), lower global cognition was found to be correlated with worse self-ratings (Cholerton et al. 2020; Pirogovsky et al. 2012), worse informant-ratings (Cholerton et al. 2020; Liepelt-Scarfone et al. 2013), worse mixed self-informant ratings (Choi et al. 2019; de Oliveira et al. 2020), as well as more errors in performance-based ADL assessments of everyday problems (Foster 2014; García-Nevares et al. 2020; Giovannetti et al. 2012; Holden et al. 2018; de Oliveira et al. 2020; Pirogovsky et al. 2012, 2014; Sulzer et al. 2020). An overview of the presented results, along with the type of assessments used, is provided in Supplementary Tables 1 and 2.

Among the range of cognitive domains, attention/working memory, executive function, and memory appear to have received the greatest attention in research, with relatively less research being published on visuo-cognitive skills and, in particular, language. Lower attention, executive functions, and memory may be related to worse self-ratings, worse informant-ratings, and worse mixed ratings of ADL function (Becker et al. 2023; Cholerton et al. 2020; Fellows and Schmitter-Edgecombe 2019; Pirogovsky et al. 2012; Puente et al. 2016). Analogously, these cognitive domains are also related to performance-based ADL assessments in people with PD, as well as visuo-cognitive skills and, although with mixed support, language function (Beyle et al. 2018; Fernández-Baizán et al. 2022; Foster and Doty 2021; Higginson et al. 2013; Giovannetti et al. 2012; Holden et al. 2020; Glonnegger et al. 2016; Manning et al. 2012; de Oliveira et al. 2020; Pirogovsky et al. 2012; Lopez et al. 2019; Puente et al. 2016; García-Nevares et al. 2020; Schmitter-Edgecombe et al. 2024). Interestingly, those patients who report spending less time per day on complex ADL tasks, indicating that they avoid or practice those challenging tasks less often, also show poorer attention and visuo-cognitive performance (Fernández-Baizán et al. 2022). In PD-MCI, attention, executive function, and memory have been found to be associated with both subjective ADL function and performance-based ADL function (Foster et al. 2022; Schmitter-Edgecombe et al. 2022; Sumida et al. 2021). Furthermore, driving performance may be primarily supported by attention, executive function, and visuo-cognitive skills (Crizzle et al. 2012; Ranchet et al. 2012). Taken together, the evidence indicates that ADL function may be supported by a broad spectrum of cognitive domains, with attention, executive function, and memory taking precedence. However, these results must be interpreted in the context of the imbalance in the literature to the detriment of visuo-cognitive skills and language.

In addition to these examinations of the relationship between ADL and cognition, several approaches have been employed to dichotomize samples based on their ADL performance. For instance, Becker et al. (2020) defined a cut-off that identified people with PD-MCI who exhibited greater cognitive than motor ADL impairment in a mixed self-informant reported ADL measure using a regression-based approach. Those scoring above the cut-off demonstrated poorer performance in both attention/working memory and language. Liepelt-Scarfone et al. (2013) found that in a cognitively mixed sample, people with PD who were reported by their informants to be more ADL impaired, performed worse in tests assessing visuo-construction and attention. Manning et al. (2012) found that non-demented people with PD who scored below 80% in a performance-based medication assessment tended to perform worse in tests assessing memory, attention, and executive function. These findings specifically highlight the role of attention/working memory in ADL function, but further research is required to establish cut-off scores.

Within the domains of attention/working memory, executive function, and memory, some functions appear to be particularly relevant for people with PD to perform their ADL adequately. Attentional performance, in particular vigilance and focused attention, is one of the strongest predictors of ADL function in PDD (Bronnick et al. 2006). In addition, the contributions of processing speed and working memory have been repeatedly emphasized (Beyle et al. 2018; Bronnick et al. 2006; Cholerton et al. 2020; Foster and Doty 2021; Fernández-Baizán et al. 2022; Glonnegger et al. 2016; Higginson et al. 2013; Manning et al. 2012). In terms of executive function, initiation, generation, and set-shifting have been highlighted as crucial skills for ADL performance (Cholerton et al. 2020; de Oliveira et al. 2020; García-Nevares et al. 2020; Glonnegger et al. 2016; Foster and Doty 2021; Lopez et al. 2019; Puente et al. 2016; Foster et al. 2022; Manning et al. 2012). Performance in immediate and delayed recall as well as content memory tasks have also been found to be stronger linked to ADL performance than other memory functions (Fellows and Schmitter-Edgecombe 2019; Sumida et al. 2021).

Given that not only memory, but also attention/working memory and executive function could potentially be the core cognitive domains to contribute to ADL performance, it seems worthwhile to consider the cognitive mechanisms that enable adequate performance of ADL tasks. One potential cognitive mechanism that modulates ADL function may be cognitive control, which relies on both attention and executive function (Diamond 2013; Hofmann et al. 2008; Miller and Wallis 2009). Cognitive control enables individuals to proactively form internal representations of goals and action plans and to maintain them in working memory (Hofmann et al. 2008). More specifically, cognitive control may play a pivotal role in the home-based environment for focusing on relevant tasks and maintaining intentions on actions amidst distractions (Buckley et al. 2014). Additionally, cognitive control allows individuals to resolve internal conflicts through the inhibition of competing interests, of temptations, and of impulses, which is also known as interference control (Aron 2011). Hence, cognitive control may play a major role in the selection, initiation, maintenance, and monitoring of ADL tasks in PD, and therefore ADL function.

### Cognition and performance-based ADL assessments

The ADL domains in performance-based assessments may differ in their demands and levels of difficulty and therefore call for the use of different cognitive functions. For example, medication management and financial administration may place greater demands on executive function, attention, and memory, but place lower demands on visuo-cognitive skills and language than other ADL domains (Lopez et al. 2019; Foster and Doty 2021; Manning et al. 2012; Sulzer et al. 2020; Pirogovsky et al. 2012). Even within the same ADL domain, tasks may vary in their cognitive requirements. For example, household chores like vacuuming and folding laundry may engage different aspects of working memory (García-Nevares et al. 2020). Thus, different cognitive functions are addressed depending on which ADL domains are assessed, which could also explain why support for the role of language in performance-based ADL function is controversial (Glonnegger et al. 2016; Sumida et al. 2021). As most studies have been conducted in cognitively mixed PD cohorts, future studies investigating the relationship between cognition and ADL function in clearly defined cognitive subgroups (e.g. PD-MCI, PDD vs. normal cognition) are needed.

Apart from these findings, qualitative analyses have revealed behavioral peculiarities in the performance of ADL tasks, suggesting that cognitive ADL impairments may involve not just errors themselves, but are also characterized by a specific abnormal behavior. Cognitively impaired people with PD seem to behave inefficiently, exhibiting aimless searching (e.g., searching through the wrong cabinet), repeating actions (e.g., towel a dry dish), stopping mid-action to correct themselves (e.g., reaching for the wrong object at first, then taking the right one; known as perplexity errors), executing tasks in the wrong order, using objects and tools in wrong positions, or perseverating on certain tasks (e.g., excessive wiping of a countertop; Beyle et al. 2018; Giovannetti et al. 2012; Glonnegger et al. 2016; Schmitter-Edgecombe et al. 2022, 2024). This trial-and-error behavior may become more pronounced as cognitive impairment worsens, since people with PDD are more likely to make perplexity errors than non-demented people with PD (Beyle et al. 2018; Glonnegger et al. 2016). In PDD, ADL impairment may further expand to omission errors, where necessary task steps are skipped (Beyle et al. 2018; Glonnegger et al. 2016). Thus, the evidence indicates that cognitive ADL impairment may be indicative of a specific behavioral pattern that can be described in terms of inefficiency, trial-and-error behavior, and, in the case of PDD, task step omissions. Considering the laboratory test situation of performance-based ADL assessments, future studies should investigate how these behavioral patterns manifest in everyday life.

## Novel methods to measure everyday behavior in PD

Within the surge of novel methods that enable the quantification of unsupervised behavior in everyday life, clinicians may have increased opportunities in making more informed decisions while minimizing economic resources. This is important because comparisons between supervised and unsupervised assessments have shown that laboratory performance is not identical to everyday behavior in people with PD (Corrà et al. 2021; Morgan et al. 2022; Rehman et al. 2022) raising questions about the transferability of laboratory ADL assessments to everyday behavior. Sensor-based methods may offer a solution to this problem, as they can capture impromptu movements and behavioral fluctuations in an unsupervised environment, thus providing naturalistic data. Because sensor-collected data are derived from behaviors in everyday life situations, which also represent ADL to a large extent (Alberdi Aramendi et al. 2018; Warmerdam et al. 2020), sensor-based methods may objectively and unobtrusively assess ADL function in the home-based environment (referring to any unsupervised setting in the home and in daily life; Warmerdam et al. 2020). Depending on the sensor type or the processing algorithm used, sensor-collected data can be translated into different parameters, such as physical activity, rest-activity rhythms, or in-home behavior. The following section will present results on the associations between sensor-based parameters, cognition, and ADL measures.

A commonly used sensor-based method to assesses everyday behavior is provided by *accelerometers*, usually worn around the hip, ankle, or wrist. Accelerometers can capture a broad range of spatial-temporal parameters of bodily behaviors, and depending on the algorithm used, can be processed to different constructs such as physical activity. Physical activity subsumes information about bodily behaviors such as sitting and lying (sedentary behaviors) or walking and standing (active behaviors; Mathie et al. 2004). Thereby, the frequency, the duration, and the metabolic demands of the bodily behaviors can be assessed, which makes it also possible to assess the pattern of physical activity (Mc Ardle et al. 2023).

Lower levels of physical activity have been linked to cognitive impairment, with the lowest levels observed in people with PDD, followed by those with PD-MCI compared to PD-NC (Cerff et al. 2017; van Uem et al. 2018). Additionally, lower levels of physical activity have been associated with self-rated ADL impairment in a cognitively mixed PD cohort (Terashi et al. 2017), and, although insignificantly, with informant-rated ADL impairment in a PDD cohort (Mc Ardle et al. 2020). Furthermore, lower global cognition, attention, and executive function have been linked to lower physical activity levels in cognitively mixed PD cohorts (Cerff et al. 2017; Donahue et al. 2022; Lamont et al. 2015; Dontje et al. 2013; Loprinzi et al. 2018; Terashi et al. 2017). A study reported that people with PD-MCI who received cognitive training increased their physical activity in comparison to an active control group, as measured by accelerometry (Bode et al. 2023). These increases were associated with improvements in executive function, indicating that those people with PD-MCI who were more physically active after the cognitive training also showed better performance in tests assessing executive functions (Bode et al. 2023). Emphasis has also been placed on the pattern of sedentary behavior. It was observed that interruptions of sedentary behaviors were less frequent in a small sample of people with PDD, who had subjectively rated ADL impairment, compared to people with PD-MCI (Cerff et al. 2017). Similarly, this sedentary pattern, indicating that sedentary bouts are more prolonged, has been linked to self-rated ADL impairment in non-PD cohorts (Chen et al. 2016; Sardinha et al. 2015; Tieges et al. 2015; Son et al. 2024). However, in people with PD-MCI, informant-rated ADL impairment was associated with a higher number of sedentary bouts (Bode et al. 2023). Interestingly, in the same cohort, prolonged sedentary bouts were associated with lower working memory (Bode et al. 2023), and prolonged sedentary bouts occurred more frequently in people with PDD than in non-demented PD (Cerff et al. 2017; van Uem et al. 2018). As explained further above, reduced physical activity could be partially due to motor-cognitive mechanisms (Ferrazzoli et al. 2018; Raffegeau et al. 2019). Accordingly, the ability to execute sit-to-stand transfers is related to self-reported ADL function in PD (Bryant et al. 2020), and falls frequently occur during sit-to-stand-transfers (Allen et al. 2013; Ashburn et al. 2008). Thus, lower physical activity levels and prolonged sedentary bouts could indicate a subjective protective strategy, where patients only get up when it is necessary (e.g. Cerff et al. 2017).

Besides physical activity, spatial-temporal parameters of bodily behaviors can also be processed to *rest-activity rhythms* (also referred to as „Actigraphy “). Rest-activity rhythms complement physical activity because they characterize the distribution and fragmentation of behaviors within a day (“intraday variability”) as well as the stability of behaviors between days (“Inter-day stability”; Gao et al. 2023). In cognitively normal people with PD, lower inter-day stability of physical activity was linked to lower attention, executive function, and visuo-cognitive skills (Leonard 2016; Wu et al. 2018). Additionally, greater intraday variability and lower inter-day stability were linked to cognitive impairment in non-PD cohorts (Rabinowitz et al. 2022). Lower inter-day stability was also reported to correlate with subjective ADL impairment in non-PD older adults with dementia, which needs to be confirmed in PD (Ishimaru et al. 2021; Carvalho-Bos et al. 2007). Overall, the evidence indicates that lower cognition and ADL impairment in PD are characterized by lower physical activity levels and prolonged sitting, and potentially a greater variability of rest-activity between and within days. However, studies are needed to examine which activities the patients engage in during these behaviors.

An alternative to accelerometers is provided by *smart-home sensors*, which can be attached to different objects within an individual’s home, such as kitchen gadgets, a washing machine, or doors. As these objects may be part of ADL scenarios, the field of smart-home research considers smart-home sensor data as direct estimate of an individual’s ADL function within their home (Yamasaki and Kumagai 2021). Different aspects of in-home behavior have been shown to reflect subjective ratings of ADL function (Alberdi et al. 2018) and performance-based ADL assessments (Suchy et al. 2020). Data obtained by pill box sensors have revealed that cognitively impaired non-PD older adults forget their medication more frequently in everyday life than cognitively normal non-PD older adults (Rawtaer et al. 2020), and medication errors have been linked to lower executive functions (Insel et al. 2006; Suchy et al. 2020). Additionally, cognitively impaired non-PD older adults tend to move less in their homes than cognitively normal non-PD older adults (Rawtaer et al. 2020; Kaye et al. 2014), to leave their homes less frequently (Wettstein et al. 2015), and to execute their ADL tasks irregularly within a day and between days (Urwyler et al. 2017). Having been interpreted to reflect disrupted and erratic routines (Lee et al. 2020), behavioral variability associated with cognitive impairment has been also reported for in-home movement, room transitions (Schütz et al. 2019), computer use (Kaye et al. 2014), walking speed (Hayes et al. 2008), and sensor patterns (Botros et al. 2022). To date, no study using smart-home sensors in people with PD could be identified. However, smart-home sensors seem to be a promising approach to get a deeper insight into patients’ home-based everyday live.

## Treatment of ADL impairment associated with cognition

As the literature suggests that cognitive ADL function offers valuable insight into the integrity of cognition in real-life situations, this underscores the need for appropriate interventions focused on improving or maintaining cognitive ADL function. To date, literature with ADL function as primary outcome in clinical intervention trial is sparse. We therefore present data on various ADL assessments and their potential to reflect treatment effects.

### Pharmacological treatment

No pharmacological treatments are yet available to improve both cognition and ADL function, or either alone, in PD-MCI. Current evidence is insufficient to recommend the use of memantine (Kawashima et al. 2022) and Acetylcholinesterase inhibitors (AChEIs, Baik et al. 2021; Mamikonyan et al. 2015; Sawada et al. 2018, 2021) in PD-MCI. In contrast, positive treatment effects of rivastigmine and donepezil for global cognitive state, memory, and speech have been reported in PDD (Brennan et al. 2016a; McShane et al. 2019; Matsunaga et al. 2015; Pagano et al. 2015; Rolinski et al. 2012). The effects of AChEIs treatments on ADL in PDD are controversially discussed. On the one hand, a meta-analysis supports a positive effect of donepezil compared to placebo for the improvement of ADL function (Matsunaga et al. 2015). On the other hand, no beneficial effect of AChEIs on ADL function in PDD could be identified (Pagano et al. 2015). In addition, discontinuation of AChEIs may have little or no effect on ADL status in the short term (Matsunaga et al. 2015; Parsons et al. 2021), and there are currently insufficient data to assess the long-term effect of AChEI on ADL function in people with dementia.

### Non-invasive non-pharmacological intervention

The value of non-pharmacological interventions for the treatment of cognitive impairment in PD is increasingly recognized, but their effect on cognitive ADL function has been addressed less often. Cognitive training, physiotherapy, and occupational therapy have been proven to improve cognition and motor skills in people with PD (Clarke et al. 2016; Leung et al. 2015). A meta-analysis, including eight studies (*n* = 485 people with PD) confirmed that moderate exercise interventions containing functional-task training had a positive effect on the UPDRS-II (Perry et al. 2019). It has also been suggested that virtual reality (Lina et al. 2020) and occupational therapy (Foster et al. 2021) have the potential to improve ADL function in PD, although the number of studies investigating these issues is limited.

Table 2 summarizes effects of non-pharmacological randomized controlled trials (RCTs) with an active control group and ADL as an outcome. Additionally, Table 2 lists study characteristics that allow for the evaluation of these RCTs conducted with greater scientific quality for a specific treatment effect on ADL function. An analogous summary of RCTs with an active control group is provided in Supplementary Table 3. In summary, only the minority of studies with an active and passive control group defined treatment induced change in ADL as primary outcome (da Silva et al. 2023; Daley et al. 2014; Foster et al. 2013; Frazzitta et al. 2012; Pompeu et al. 2012). Effective physical RCTs with an active control group applied aerobic training (Burini et al. 2006; Schenkman et al. 2012), physical therapy (Dereli and Yaliman 2010), resistance training and fall education, movement strategy training (Morris et al. 2015; King et al. 2015), postural breathing exercises (Paolucci et al. 2017), balance trainings (Volpe et al. 2014), and walking coordination trainings (Zhu et al. 2018). Combined interventions, consisting of multiple elements such as cognitive, motor training, occupational therapy, or psychomotor exercises, have been shown to provide benefits for ADL function **(**Sousa et al. 2021; Pedreira et al. 2013; Palamara et al. 2017), greater than either alone or than less demanding interventions (Choi and Cho 2022; Monticone et al. 2015; Pompeu et al. 2012; Tamir et al. 2007). So far, only one study revealed a positive effect of cognitive training vs. an active control group on ADL (Peña et al. 2014). Treatment duration varies between 30 (Ribas et al. 2017; Pompeu et al. 2012) and 120 (Morris et al. 2015) minutes in RCTs with active control group, and the weekly treatment frequencies are highly variable between studies. A variety of studies used the UPDRS-II as study outcome (Burini et al. 2006; Dereli and Yaliman 2010; King et al. 2015; Morris et al. 2015; Paolucci et al. 2017; Schenkman et al. 2012; Tamir et al. 2007; Volpe et al. 2014; Zhu et al. 2018; Pompeu et al. 2012) and did not correct their statistical analyses for the influence of motor impairment, albeit the UPDRS-II is confounded by such. Only few of the identified RCTs with active control group included more than 30 persons per active control group (Monticone et al. 2015; Morris et al. 2015; Schenkman et al. 2012; Choi and Cho 2022). Most important, people with PDD were excluded in nearly all non-pharmacological intervention trials (Folkerts et al. 2018), thus the evaluation of treatment effects is limited to people without dementia. To date, only some studies investigate treatment effects in homogenous groups of people with PD-MCI (Kalbe et al. 2020), so more studies are needed to evaluate treatment effects on ADL in distinct cognitive subgroups.

**Table 2:** Selected (quasi-) randomized controlled trials (RCT) with an active control group and activity of daily living (ADL) function as primary, secondary, or explorative outcome in Parkinson’s disease (PD)

Author	Design	Intervention	Age in years^1^	Disease duration (DD) in years and H&Y^a^	Treatment duration & intensity^b^	ADL outcome	Treatment effects on ADL	Comments
Physical intervention								
Burini et al. (2006)	Single-blind cross-over RCT	EG: Aerobic training (*n* = 13, DO = 2; ergometer) vs. CG: Qigong (*n* = 13, DO = 2)	All participants: 65.2 ± 6.5	DD 10.8 ± 4.6 H&Y (%) Stage 2/3: 26.9/73.1	EG: 45 min, 3 times per week for 7 weeks (total of 20 sessions); CG: 50 min, 3 times per week for 7 weeks (total of 20 sessions)	Unified Parkinson’s Disease Rating Scale part II (UPDRS-II), Brown’s Disability Scale (BDS)	No effect in UPDRS-II; no effect in BDS	Dementia (MMSE < 24) excluded, no participation in any other intervention; stable medication during trial
Dereli and Yaliman (2010)	Single-blind quasi-randomized trial (alternate allocation)	EG: Physiotherapy (*n* = 15, DO = 1) vs. CG: Unsupervised home-based exercise (*n* = 15, DO = 1)	EG: 66.5 ± 12.9 CG: 61.3 ± 9.6	DD EG: 6.3 ± 4.8 CG: 6.7 ± 2.4 H&Y EG: 2.1 ± 0.6 CG: 2.1 ± 0.7	45 min, 3 times per week for 10 weeks (30 sessions)	UPDRS-II	↑ EG vs. CG (change scores, Pre vs. Post)	Dementia (MMSE < 24) excluded; stable dopaminergic medication during trial
King et al. (2015)	Single-blind RCT, stratification by comorbidity level	EG1: Exercise targeting posture, bradykinesia, & gait coordination at home (*n* = 17, DO = 0) vs. EG2: On-site 1-to-1 variant of EG1 (*n* = 21, DO = 0) vs. CG: On-site group variant of EG1 (*n* = 20, DO = 1)	EG1:64.6 ± 6.8 EG2: 64.2 ± 6.7 CG: 63.9 ± 8.5	DD EG1: 5.2 ± 5.8 EG2: 7.9 ± 7.9 CG: 5.4 ± 3.6 H&Y EG1: 2.5 ± 0.5 EG2: 2.4 ± 0.5 CG: 2.4 ± 0.5	60 min, 3 times per week for 4 weeks (12 sessions)	UPDRS-II	↑ EG2 (change scores, Pre vs. Post)	Dementia excluded, ability to walk without assistance, no support in ADL, no participation in any other intervention
Morris et al. (2015)	Single-blind RCT	EG1: Progressive resistance training & fall education (*n* = 70, DO = 1–3) vs. EG2: Movement strategy training (*n* = 69, DO = 2–3) vs. CG: Life skills training (*n* = 71, DO = 12–14)	EG1: 67.4 ± 10.4 EG2: 68.4 ± 9.9 CG: 67.9 ± 8.4	DD EG1: 6.7 ± 5.6 EG2: 7.2 ± 6.2 CG: 6.0 ± 5.5 H&Y (%) Stage 0–1/1.5/2/2.5/3/4: EG1: 10.5/5.7/31.4/15.7/30.0/7.1 EG2: 13.4/4.3/24.6/21.7/27.5/8.7 CG: 8.4/7.0/24.0/10.0/32.4/18.3	120 min, once per week for 8 weeks (8 sessions)	UPDRS-II	↑ EG1 vs. CG (Pre vs. Follow-Up), ↑ EG2 vs. CG (Pre vs. Follow-Up)	Dementia (MMSE < 25) and DBS excluded
Paolucci et al. (2017)	Single-blind RCT	EG: Postural breathing exercises (*n* = 17, DO = 0) vs. CG: Home-based postural breathing exercises (*n* = 19, DO = 2)	Median, Q_0.75_-Q_0.25_ EG: 66.0, 18.5 CG: 67.0, 11.0	All participants: DD 3.0 ± 1.2 H&Y 1.5 ± 0.8	60 min, 2 times per week for 5 weeks (10 sessions)	UPDRS-II	↑ EG (Pre vs. Post, Post vs. Follow-Up), ↑ CG (Pre vs. Post)	Dementia (MMSE < 27) and DBS excluded; no participation in any other rehabilitation program
Schenkman et al. (2012)	Single-blind RCT, stratification by gender	EG1: Aerobic training (*n* = 41, DO = 7–10 vs. EG2: Flexibility & balance exercises (*n* = 39, DO = 3–6) vs. CG: Home-based exercises (*n* = 41, DO = 6–9)	EG1: 63.4 ± 11.2 EG2: 64.5 ± 10.0 CG: 66.3 ± 10.1	DD EG1: 3.9 ± 4.2 EG2: 4.9 ± 3.7 CG: 4.5 ± 3.8 H&Y EG1: 2.2 ± 0.5 EG2: 2.3 ± 0.4 CG: 2.3 ± 0.4	50–55 min, 5–7 times per week for 16 months (320–448 sessions); EG1 & EG2: supervision 3 times per week for 4 months, then once a month; CG: supervision once a month	UPDRS-II	↑ EG1 vs. CG (Pre vs. Post); ↑ EG1 & EG2 vs. CG (Pre vs. Post vs. Follow-Up 1 vs. Follow-Up 2)	Dementia (MMSE < 24) excluded; ability to walk without assistance
Volpe et al. (2014)	Single-blind RCT	EG: Balance training in water (*n* = 17, DO = 0) vs. CG: Land-based balance training (*n* = 17, DO = 0)	EG: 68 ± 7 CG: 66 ± 8	DD EG: 7.5 ± 5.1 CG: 7.6 ± 4.6 H&Y EG: 2.8 ± 0.3 CG: 2.7 ± 0.5	60 min, 5 times per week for 8 weeks (40 sessions)	UPDRS-II	↑ EG & CG (Pre vs. Post)	Dementia (MMSE < 24) excluded, ability to walk without assistance but ≥ 2 falls within the past 12 months
Zhu et al. (2018)	Single-blind RCT	EG: Walking coordination exercises in water (*n* = 23, DO = 0) vs. CG: Balance exercises in water (*n* = 23, DO = 0)	EG: 65 ± 6 CG: 67 ± 5	DD EG: 6.8 ± 2.6 CG: 6.7 ± 2.4 H&Y EG: 2.4 ± 0.4 CG: 2.4 ± 0.4	40 min, 5 times per week for 6 weeks (30 sessions)	UPDRS-II	↑ EG & CG (Pre vs. Post vs. Follow-Up)	Dementia (MMSE < 24) and DBS excluded, ability to walk without assistance for ≈45.7 m and stand independently, no participation in any other physical therapy within the past 6 months
**Cognitive training**								
Kalbe et al. (2020)	Single-blind RCT	EG: “NEUROvitalis” (*n* = 33, DO = 2) vs. CG: Low intensity physical activity training (*n* = 31, DO = 1)	EG: 67.7 ± 7.2 CG: 67.5 ± 8.3	DD (Median, range) EG: 7.2, 0.8–30.1 CG: 6.3, 1.5–14.5 H&Y (%) Stage 1/2/3/4: EG: 9.1/57.6/30.3/3.0 CG: 19.4/61.3/19.4/0.0	90 min, 2 times per week for 6 weeks (12 sessions)	Bayer Activities of Daily Living Scale (B-ADL)	No effect	Only participants with PD-MCI (MDS criteria); dementia (MDS criteria), depression (BDI-II ≥ 20), severe fatigue, and DBS excluded, no participation in any other treatment study within the past 2 months
París et al. (2011)	Single-blind RCT, stratification by age & premorbid intelligence	EG: “SmartBrain” (*n* = 18, DO = 2) vs. CG: Speech therapy (*n* = 15, DO = 3)	EG: 64.8 ± 9.2 CG: 65.4 ± 9.6	DD EG: NA CG: NA H&Y EG: 2.4 ± 0.8 CG: 2.3 ± 0.8	45 min, 3 times per week for 4 weeks (12 sessions)	Cognitive Difficulties Scale (CDS)	No effect	Dementia (MMSE < 24 or intake of AChEI) and depression (GDS-15 > 10) excluded, no participation in any other cognitive, psychological, speech, or physical intervention; stable medication during trial
Peña et al. (2014)	Single-blind RCT	EG: “REHACOP” (*n* = 22, DO = 2) vs. CG: Occupational therapy (*n* = 22, DO = 0)	EG: 67.6 ± 5.2 CG: 68.1 ± 7.5	DD EG: 5.5 ± 4.6 CG: 7.4 ± 5.7 H&Y (%) Stage 1/2/3: EG: 18.2/81.8/0.0 CG: 22.7/68.2/9.1	60 min, 3 times per week for 9 weeks (27 sessions)	World Health Organization Disability Assessment Schedule version II (WHODAS 2.0)	↑ EG vs. CG (change scores, Pre vs. Post)	Dementia (MDS & DSM-IV-TR criteria) and depression excluded
Sousa et al. (2021)	RCT (no information regarding blinding)	EG: cognitive training & multi-domain intervention (*n* = 24, DO = 0) vs. CG: only multi-domain intervention (*n* = 15, DO = 0)	EG: 60.0 ± 7.5 CG: 58.5 ± 9.8	DD EG: 5.7 ± 3.3 CG: 6.8 ± 8.8 H&Y: EG: 87.5% in stage 1–2 CG: 93.3% in stage 1–2	2 times per week (120 min) for 4 weeks (8 sessions)	ADL part of Parkinson's Disease Questionnaire (PDQ-39)	↑ EG (Pre vs. Post)	Only participants with PD-MCI (MDS criteria); Depression (BDI-II ≥ 16) excluded, stable response to medication during trial, no participation in other cognitive training within the past year
**Multi-domain intervention**								
Choi and Cho (2022)	Single-blind RCT	EG: Daily life training (*n* = 30, DO = 0) vs. CG: Traditional rehabilitation involving motor tasks, basic ADL tasks, & joint exercises (*﻿n* = 30, DO = 0)	EG: NA CG: NA	DD EG: NA CG: NA H&Y All participants: Stage 3	50 min, 2 times per week for 5 weeks (10 sessions)	New ADL Questionnaire	↑ EG vs. CG (change scores, Pre vs. Post), ↑ EG (Pre vs. Post)	Dementia (MMSE < 20) excluded
Monticone et al. (2015)	Single-blind RCT	EG: Training involving ergonomic education, motor & cognitive tasks (*n* = 35, DO = 2–3, analyzed = 35) vs. CG: light mobility training (*n* = 35, DO = 1–3, analyzed = 35)	EG: 74.1 ± 6.0 CG: 73.4 ± 7.0	DD EG: 1.7 ± 2.6 CG: 25.5 ± 3.8 H&Y (%) Stage 2.5/3/4: EG: 22.9/57.1/20.0 CG: 20.0/62.9/17.1	EG & CG: Physical training—90 min, 7 times per week for 8 weeks (56 sessions); EG: Cognitive training—30 min, 2 times per week for 8 weeks (16 sessions); Occupational training—30 min, once per week for 8 weeks (8 sessions)	Functional Independence Measure (FIM), ADL part of PDQ-39	↑ EG vs. CG (Pre vs. Post vs. Follow-Up) in FIM; ↑ EG vs. CG (Pre vs. Post vs. Follow-Up) in ADL part of PDQ-39	Only participants with decline in physical function; dementia (MMSE < 24), psychiatric disorders, invasive drug treatment, and DBS excluded
Palamara et al. (2017)	Single-blind RCT	EG1: Aerobic, balance, & mobility exercises in water plus “multidisciplinary intensive rehabilitation treatment” (MIRT) & occupational ADL therapy (*n* = 17, DO = 0) vs. CG: MIRT (n = 17, DO = 0)	EG: 70.9 ± 5.7 CG: 70.8 ± 5.3	DD EG: NA CG: NA H&Y EG: 2.8 ± 0.5 CG: 3.1 ± 0.2	EG: MIRT—60 min, 4 times per day for 5 days per week for 4 weeks (100 sessions); Aquatic therapy—45–60 min, 3 times per week for 4 weeks (12 sessions); CG: MIRT—60 min, 4 times per day for 5 days per week for 4 weeks (100 sessions)	UPDRS-II	↑ EG & CG (Pre vs. Post)	Dementia (MMSE < 24) & DBS excluded
Pedreira et al. (2013)	Single-blind RCT	EG: Exergaming (n = 22, DO = 6; Wii-Fit games) vs. CG: Physical therapy (n = 22, DO = 6)	EG: 61.1 ± 8.2 CG: 66.2 ± 8.5	DD EG: 8.6 ± 4.6 CG: 7.3 ± 6.6 H&Y EG: 2.5 ± 0.6 CG: 2.4 ± 0.7	50 min, 3 times per week for 4 weeks (12 sessions)	ADL part of PDQ-39	↑ EG (Pre vs. Post)	Dementia and psychiatric disorders excluded
Pompeu et al. (2012)	Single-blind RCT	EG: Exergaming (n = 16, DO = 0; Wii-based motor-cognitive training) vs. CG: Balance training (n = 16, DO = 0)	EG: 68.6 ± 8.0 CG: 66.2 ± 8.3	DD EG: 4.7 ± 5.4 CG: 5.2 ± 3.4 H&Y EG: NA CG: NA	30 min global exercises (e.g. stretching, strengthening) & 30 min group-specific training, 2 times per week for 7 weeks (14 sessions)	UPDRS-II (primary outcome)	↑ EG & CG (Pre vs. Post, Post vs. Follow-Up)	Dementia (MMSE < 24) & depression (GDS-15 ≥ 6) excluded; no prior experience with Wii Fit; no participation in any other intervention
Ribas et al. (2017)	Single-blind pilot RCT	EG: Exergaming (n = 10, DO = 0; Wii-Fit games) vs. CG: Conventional exercise including stretching & resistance exercises for the limbs (n = 10, DO = 0)	EG: 61.7 ± 6.8 CG: 60.2 ± 11.3	DD EG: 6.5 ± 4 CG: 7 ± 2.8 H&Y (Median, IQR) EG: 1.3, 1–2 CG: 1.5, 1–2	30 min, 2 times per week for 12 weeks (24 sessions)	ADL part of PDQ-39	No effect	Dementia (MMSE < 24) excluded; no prior experience with Wii balance board; no participation in any other intervention
Tamir et al. (2007)	Single-blind RCT, stratification by age, gender, & disease stage	EG: Combination of imagery & physical practice (n = 12, DO = 1) vs. CG: Physical practice (n = 11, DO = 1; same motor task as EG)	EG: 67.4 ± 9.7 CG: 67.4 ± 9.1	DD EG: 7.4 ± 3.1 CG: 7.8 ± 4.5 H&Y EG: 2.3 ± 0.4 CG: 2.3 ± 0.4	60 min, 2 times per week for 12 weeks (24 sessions)	UPDRS-II, Schwab & England ADL Scale (S&E-ADL)	No effect in UPDRS-II; ↑ EG vs. CG (Pre vs. Post) in S&E-ADL	Dementia (MMSE < 25) excluded; stable medication during trial

^a^. If not otherwise indicated, values given as Mean ± SD, ^b^. If not otherwise indicated, information applies to both EG and CG, *AChEI* Acetylcholinesterase inhibitors; *BDI-II* Beck Depression Inventory II; *CG* Control group; *DBS* Deep brain stimulation; *DD* Disease duration; *DO*. Drop-out; *DSM-IV-TR* Diagnostic and Statistical Manual of Mental Disorders 4th Edition; *EG* Experimental (treatment) group; *GDS-15* Geriatric Depression Scale; *H&Y* Hoehn & Yahr stage; *m* Meter; *min* Minute; *MDS* Movement Disorder Society; *MMSE* Mini Mental State Exam; *NA* Not available; *PD-MCI* PD with mild cognitive impairment

Interventional studies in non-PD older adults with MCI may offer insights into the positive impact of ADL trainings as a future directive to prevent further ADL decline in the prodromal phase of PDD, namely PD-MCI. These studies demonstrate that interventions, such as cognitive trainings, cognitive rehabilitation, multi-domain trainings, and everyday problem-solving strategy trainings improve ADL performance compared to control groups (Schmitter-Edgecombe and Dyck 2014; Park 2022; Chandler et al. 2016; Tulliani et al. 2022). Moreover, the training impact is not limited to the improvements in ADL performance but also positively affects cognitive functioning, especially executive and memory function (Park 2022; Schmitter-Edgecombe et al. 2022). Therefore, findings in non-PD cohorts suggest that an ADL training may effectively improve IADL function of people with PD-MCI. Home-based digital CTs are increasingly tested as innovative strategies to improve cognition in patients with limited access to healthcare facilities due to disabilities such as traveling difficulties (Biundo et al. 2017). Digital CTs have been shown to improve cognition as assessed by cognitive tests (Gavelin et al. 2022), but their positive effect on ADL either short- or long-term and, accordingly, its potential to prevent PDD has not been investigated yet.

## Discussion and conclusion

**Fig. 1:**
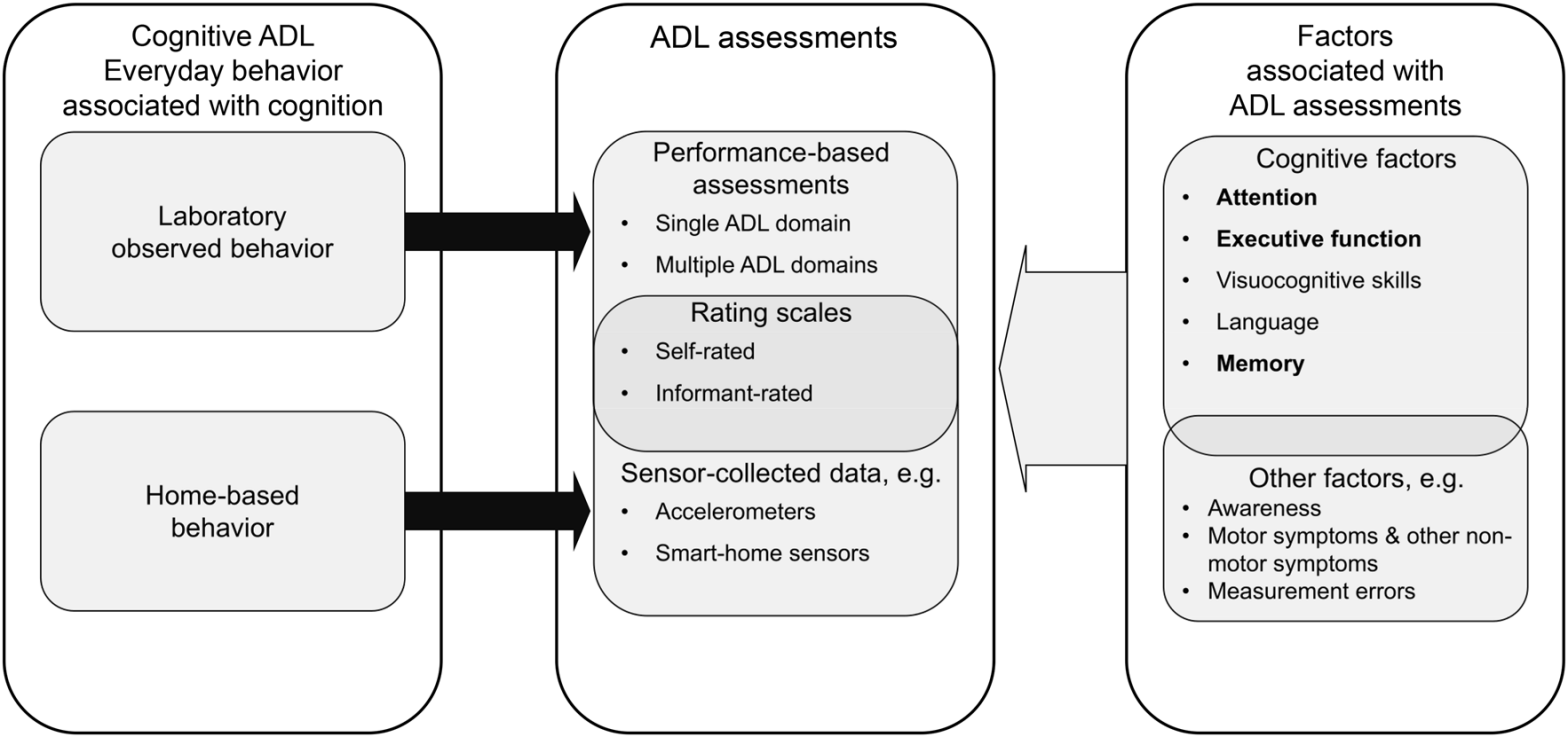
This figure depicts the conceptualization of cognitive ADL. Cognitive ADL function refers to the functionality in everyday behaviors that are associated with cognition. These behaviors can be observed in a laboratory environment, measured with performance-based ADL assessments, or measured in the home-based environment (referring to any unsupervised setting in the home and in daily life) with sensor-collected data. Rating scales may represent an overlap of these two observational situations, as raters may draw from their observations in everyday life to respond to a laboratory assessment. Different factors, which partially overlap, may be associated with these ADL assessments, including cognitive factors (with attention, executive function, and memory being particularly important) and other factors

ADL function can be viewed as a multifaceted construct that reflects functionality in different daily life situations, and therefore provides clinicians comprehensive insight into their patient’s overall health and well-being. The assessment of ADL function is crucial for cognitive diagnosis: The core feature that differentiates PDD from PD-MCI is the loss of the ability to perform ADL tasks that are necessary for independent living due to cognitive deterioration (Dubois et al. 2007). Because motor and non-motor symptoms of people with PD can impact their ADL function, clinicians should carefully assess the primary factor that contributes to a patient’s ADL impairment, as far as possible. Assessing ADL tasks that are primarily reliant on cognition could enhance the precision of diagnostic procedures because these tasks indicate how cognitive skills are used in real-world scenarios embedded within a context-rich environment. To that end, however, more systematic examinations are required, for which a proper nomenclature for the multifaceted construct of ADL is necessary. Therefore, we here introduce the concept of cognitive ADL impairment (see Fig. 1), which reflects problems in everyday life due to cognitive deterioration.

Impairment in IADL tasks is reported in about 30% to 50% of people with PD-MCI (Glonnegger et al. 2016; Martin et al. 2013). Importantly, those people with PD-MCI who display exclusive impairment in IADL that place greater demands on cognition are at an increased risk to develop PDD within three years (Becker et al. 2022a). Therefore, people with PD-MCI and cognitive ADL impairment should be considered an important target group for pharmacological and non-pharmacological interventions that maintain or improve their everyday life.

Currently, we face several challenges in research on effective interventions targeting cognitive ADL impairment. Firstly, the diagnostic properties of clinical instruments to assess cognitive ADL impairment are still insufficiently explored and existing data need to be verified in larger samples. It must be acknowledged that the views of patients and their informants may be confounded by several biases, with particular caution regarding those biases conflicting with the assessment of cognitive dysfunction. Sensor-based assessments could offer a novel approach to objectively assess ADL function in terms of manifest behavior. Nevertheless, ADL rating scales offer the advantage that they synthesize multiple observations from everyday life over an extended time period in comparison to the patient’s premorbid ability. Therefore, the results of subjective ADL assessments may be of value for the screening of everyday problems, which practitioners should then explore in detail in a patient- and/or caregiver-centered interview. Secondly, knowledge of the time course of cognitive ADL impairment in people with PD is sparse. Prospective longitudinal studies that examine the onset and the severity of ADL impairment in the prodromal phase of PDD are urgently needed to understand the causal relationship between ADL impairment and cognitive worsening.

Our construct of cognitive ADL function may provide a framework for research that aims to enhance the accuracy of diagnostic methods. While new ADL assessments could be developed, existing ADL scales could be extended or adapted as well. For example, total scores for cognitive ADL function might only include cognitive ADL items, or adjusted cognitive ADL scores may weight each ADL item depending on their cognitive demands. Another approach could be to modify items of existing scales to ask for the primary cause for a patient’s difficulties. First evidence supports that informants attribute different sources to the patient's difficulties in an ADL task (Benge and Balsis 2016). Nevertheless, it remains a challenge to disentangle patients’ and caregivers’ ADL ratings from the different biases affecting their responses (e.g., Caap-Ahlgren and Dehlin 2002; Ransmayr 2020). More studies are needed to get further insight how to minimize the biases of ratings of everyday skills.

Although a broad range of cognitive functions may be implicated in the ADL function of people with PD, attention, executive functions, and memory are most consistently highlighted in the current body of research (Holden et al. 2020; Sulzer et al. 2020; Cholerton et al. 2020). However, future research must strive to equilibrate the imbalance in literature for the examination of the role of language and visuo-cognition. Several mechanisms, such as cognitive control or mechanisms associated with memory, may be at play in the establishment of these relationships. Longitudinal studies and experimental paradigms are necessary to understand the specific cognitive mechanisms that link cognition and ADL function. With such an understanding, it will be possible to provide effective interventions that are precisely tailored to the specific causes of a patient’s cognitive ADL impairment, thereby improving patient-centered medical care.

Based on studies that have used performance-based ADL assessments, it can be inferred that ADL impairment associated with cognition may be characterized by a specific error profile (Beyle et al. 2018; Glonnegger et al. 2016; Schmitter-Edgecombe et al. 2022). Future studies using smart-home sensors might lead to a deeper understanding of patients’ everyday behavior, but such studies are not yet available. As performance-based ADL assessments are time consuming and involve a high level of material expenditure, digital assessments targeting the characteristic behavior of people with PD might be helpful to detect cognitive ADL impairment at its early stage.

Therefore, monitoring the home-based behavior of people with PD might be a future directive to get an ecologically valid insight into patients’ everyday life. On the one hand, cognitive ADL impairment may be characterized by lower engagement in everyday behaviors, as suggested by physical activity levels, prolonged sedentary behavior, and in-home behavior (Terashi et al. 2017; Cerff et al. 2017; Rawtaer et al. 2020). On the other hand, cognitive ADL impairment may be characterized by a high variability within and between days, which is impossible to detect with one-time clinical assessments (Urwyler et al. 2017; Ishimaru et al. 2021). More effort is warranted to understand the relationship between these behavioral aspects and their subjective experience by patients and informants with ADL function and cognition, as there appears to be a gap between subjective and objective measures (Foster and Hershey 2011; Puente et al. 2016; Vlagsma et al. 2017).

Physical, cognitive, and multi-domain interventions can improve ADL function in PD. Unfortunately, the ADL measures used as outcomes in currently available RCTs are often confounded by motor performance (Lee et al. 2014; Skinner et al. 2015; Rosenthal et al. 2010). Further non-pharmacological blinded RCTs in large multicenter studies with cognitive ADL function as the primary outcome are needed to fully recognize the benefit of preventing or delaying the worsening of cognitive ADL impairment in the prodromal phase of PDD. These interventions should be specifically targeted on cognitive ADL function, as this may be an even more important outcome than laboratory-assessed cognition. Cognitive ADL tasks often involve open-ended activities in distracting environments, unlike the structured cognitive tests conducted in quiet laboratory conditions. Additionally, cognitive ADL tasks might require multiple cognitive functions simultaneously, contrasting with traditional cognitive tests which assess one cognitive function at a time. Most importantly, people with PD have a strong desire to maintain their autonomy and their self-reliance (Haahr et al. 2011), while dreading becoming dependent on others and burdening their loved ones (Vann-Ward et al. 2017). Therefore, enhancing cognitive ADL function aligns directly with meeting the patients' needs. In this regard, home-based digital trainings offer an innovative and self-reliance-promoting strategy to improve cognition and cognitive ADL function in patients with difficulties in travelling, which limits patients’ access to healthcare facilities (Zaman et al. 2021).

In conclusion, there is a great need for ADL assessments and ADL treatments specifically designed to target cognitive ADL impairment in PD. Early detection and treatment of cognitive ADL impairment shows great potential to delay or prevent dementia conversion in people with PD and enhancing the quality of life for patients and caregivers.

## Supplementary Information

Below is the link to the electronic supplementary material.

Supplementary file1 (DOCX 96 KB)

## Data Availability

Data sharing is not available for this article as no original datasets were generated or analyzed for this narrative review.
