# Supplementary material for: Cognition and Activity of Daily Living Function in people with Parkinson’s disease
Source: J Neural Transm (Vienna). 2024 Jul 8;131(10):1159–86. doi: 10.1007/s00702-024-02796-w (PMC11489248; doi:10.1007/s00702-024-02796-w)
Supplement: Supplementary file 1 — Supplementary file1 (DOCX 96 KB) [file 702_2024_2796_MOESM1_ESM.docx]

**Supplementary Table 1.** Selected studies associating the strength of cognition and (instrumental) activity of daily living (IADL) function assessed with self-report and informant-report in Parkinson’s disease (PD)

| **Author** | **Covariates** | **Cohort** | **ADL measure** | **Global cognition** | **Executive function** | **Attention and working memory** | **Memory** | **Visuo-cognitive skills** | **Language** |
| --- | --- | --- | --- | --- | --- | --- | --- | --- | --- |
| **Self-report** | | | | | | | | | |
| Becker et al. (2023) | NA | Non-PDD;  *N* = 74 | Lawton & Brody IADL | / | Semantic Fluency (animals) | / | / | / | / |
| Cholerton et al. (2020) | NA | Mixed;  *N* = 192 | Parkinson’s Daily Activities Questionnaire–15 (PDAQ-15) | MoCA | Semantic Fluency (animals) &  TMT B – A | Letter-Number Sequencing (WAIS-III) & Digit-Symbol Test (WAIS-R) | HVLT-R (immediate & delayed recall) | n.s. | / |
| Fellows and Schmitter-Edgecombe (2019) | NA | Non-PDD;  *N* = 35 | Instrumental Activities of Daily Living – Compensation Scale (IADL-C) | / | / | / | Activity Memory (content memory & temporal order) | / | / |
| Fernández-Baizán et al. (2022) | NA | MoCA ≥ 22;  *N* = 40 | Activity Record of National Institute of Health (ACTRE) | / | / | Digit Span Backward (WMS-III), Spatial Span Forward & Backward (CANTAB) | / | Benton Judgement of Line Orientation (brief version) | / |
|  |  | MoCA ≤ 21;  *N* = 15 | ACTRE | / | / | Spatial Span Forward (CANTAB) | n.s. | n.s. | / |
| Lopez et al. (2019) | NA | MMSE > 24; *N* = 19 | Schwab and England Scale (S&E-ADL) | / | / | / | / | / | / |
| Pirogovsky et al. (2012) | NA | Mixed;  *N* = 33 | Medication Management Efficacy Scale (MMES) | MDRS total score | / | / | MIST | / | / |
|  |  |  | Lawton & Brody IADL | n.s. | / | / | MIST | / | / |
| Schmitter-Edgecombe et al. (2022) | NA | PD-MCI;  *N* = 15 | IADL-C | / | n.s. | Attention domain score | n.s. | / | / |
| **Informant-report** | | | | | | | | | |
| Fellows & Schmitter-Edgecombe (2019) | NA | Non-PDD;  *N* = 37 | IADL-C | / | / | / | Activity Memory (content memory) | / | / |
| Cholerton et al. (2020) | NA | Mixed;  *N* = 192 | PDAQ-15 | MoCA | Semantic Fluency (animals) &  TMT B - A | Letter-Number Sequencing (WAIS-III) & Digit-Symbol Test (WAIS-R) | HVLT-R (immediate & delayed recall) | n.s. | / |
| Puente et al. (2016) | NA | Undefined (MDRS-2 total score range = 115-144);  *N* = 85 | Lawton & Brody IADL | MDRS-2 total score | TMT B - A & Brixton SAT | / | / | / | / |
| **Mixed Self- & informant report** | | | | | | | | | |
| Choi et al. (2019) | *Age, H&Y, UPDRS II, III, IV motor fluctuations, BDI, & NMSS | Non-PDD;  *N* = 106 | Korean IADL (K-IADL) | MMSE* | / | / | / | / | / |
| de Oliveira et al. (2020) | NA | Mixed (suspected mild PDD);  *N* = 32 | Pfeffer Functional Activities Questionnaire (FAQ) | MMSE &  MDRS total score | / | / | / | / | / |

Legend: BDI. Beck Depression Inventory, Brixton SAT. Brixton Spatial Anticipation Test, CANTAB. Cambridge Neuropsychological Test Automated Battery, HVLT-R. Hopkins Verbal Learning Test-Revised, H&Y. Hoehn & Yahr Scale, MDRS(-2). Mattis Dementia Rating Scale (2), MIST. Memory for Intentions Screening Test, Mixed. sample includes Non-PDD and potentially PDD, MMSE. Mini Mental State Exam, MoCA. Montreal Cognitive Assessment, NA. Not available, NMSS. Non-Motor Symptoms Scale for Parkinson’s disease, Non-PDD. dementia was exclusion criterion, n.s. not significant, PDD. Parkinson’s disease dementia, PD-MCI. Parkinson’s disease with mild cognitive impairment, TMT. Trail Making Test, TMT B - A. Trail Making Test part B minus part A, UPDRS II/III/IV. Unified Parkinson’s Disease Rating Scale part II/ part III/ part IV, WAIS-III/-R. Wechsler Adult Intelligence Scale-Third Edition/ -Revised, WMS-III. Wechsler Memory Scale-Third Edition, /. not assessed, *. corrected for covariates listed in covariates column

**Supplementary Table 2.** Selected studies associating the strength of cognition and (instrumental) activity of daily living (IADL) function assessed with performance-based assessments in Parkinson’s disease (PD)

| **Author** | **Covariates** | **Cohort** | **ADL measure** | **Global cognition** | **Executive function** | **Attention and working memory** | **Memory** | **Visuo-cognitive skills** | **Language** |
| --- | --- | --- | --- | --- | --- | --- | --- | --- | --- |
| Beyle et al. (2018) | NA | Mixed;  *N* = 73 | Multiple Object Test (MOT; total errors) | / | n.s. | TMT-A | Constructional Praxis Recall (CERAD) | Object Decision (VOSP) | n.s. |
| Foster (2014) | NA | Non-PDD diagnosis (MMSE ≥ 25);  *N* = 77 | Performance Assessment of Self-care Skills (PASS; total score) | MMSE | / | / | / | / | / |
|  |  |  | PASS (money management) | MMSE | / | / | / | / | / |
|  |  |  | PASS (medication management) | MMSE | / | / | / | / | / |
| Foster and Doty (2021) | NA | Non-PDD diagnosis (MMSE ≥ 25);  *N* = 102 | PASS (total score) | / | Intra-Extra Dimensional Set Shift Test (CANTAB) & Stockings of Cambridge (CANTAB) | Spatial Span (CANTAB) | Paired Association Learning Test (CANTAB) | / | / |
|  |  |  | PASS (medication management) | / | n.s. | Spatial Span (CANTAB) | Paired Association Learning Test (CANTAB) | / | / |
| Fernández-Baizán et al. (2022) | NA | MoCA ≥ 22;  *N* = 40 | Assessment of Motor and Process Skills (AMPS; L1: fold a basket of clean clothes) | / | / | / | Digit Span Backward (WMS-III) | n.s. | / |
|  |  |  | AMPS (J4: vacuum, move light furniture) | / | / | Spatial Span Forward (CANTAB) | n.s. | Benton Judgement of Line Orientation (brief version) | / |
|  |  |  | AMPS (F4: grilled cheese sandwich and drink) | / | / | n.s. | n.s. | n.s. | / |
|  |  |  | AMPS (D2: scrambled or fried eggs, toast, and coffee or tea) | / | / | Spatial Span Forward (CANTAB) | n.s. | n.s. | / |
|  |  | MoCA ≤ 21;  *N* = 15 | AMPS (L1, J4, F4) | / | / | Spatial Span Backward (CANTAB) | / | n.s. | / |
|  |  |  | AMPS (D2) | / | / | Spatial Span Backward (CANTAB) | / | Benton Judgement of Line Orientation (brief version) | / |
| García-Nevares et al. (2020) | *Age & gender | Mixed;  *N* = 64 | AMPS (L1) | MoCA* | Semantic Fluency (EVOCAT)* | / | / | / | / |
|  |  |  | AMPS (F4) | n.s. | Semantic Fluency (EVOCAT)* & Stroop Interference* | / | / | / | / |
| Giovannetti et al. (2012) | NA | Mixed;  *N* = 40 | Naturalistic Action Test (NAT; omission errors) | MMSE | Executive control domain score | / | Episodic memory domain score | / | / |
|  |  |  | NAT (commission errors) | MMSE | Executive control domain score | / | Episodic memory domain score | / | / |
| Glonnegger et al. (2016) | NA | Mixed;  *N* = 133 | MOT (total errors) | / | TMT-B, Figure Test (NAI), Tower of London, Berlin Apraxia Test (NAI), & Verbal Fluency (CERAD) | Alertness (TAP), Go-Nogo (TAP), TMT-A, Digit Span Forward & Backward (WMS-R) | Constructional Praxis Recall (CERAD) | Constructional Praxis (CERAD), Object Decision (VOSP), & Wordlist (memory, recall, & recognition; CERAD) | / |
| Higginson et al. (2013) | * Age, GDS, & UPDRS III | Non-PDD;  *N* = 30 | Timed IADL (TIADL) | / | TMT (Number-Letter Switching) | TMT (Letter Sequencing)* & TMT (Visual Scanning, Number Sequencing, & Letter Sequencing) | / | / | / |

**[Continued] Supplementary Table 2.** Selected studies associating the strength of cognition and (instrumental) activity of daily living (IADL) function assessed with performance-based assessments in Parkinson’s disease (PD)

| **Author** | **Covariates** | **Cohort** | **ADL measures** | **Global cognition** | **Executive function** | **Attention and working memory** | **Memory** | **Visuo-cognitive skills** | **Language** |
| --- | --- | --- | --- | --- | --- | --- | --- | --- | --- |
| Holden et al. (2018) | * age, education, disease duration, & LEDD | PD-MCI;  *N* = 19 | University of California San Diego Performance-Based Skills Assessment (UPSA) | MDRS-2 total score* | / | / | / | / | / |
|  |  | PDD;  *N* = 25 | UPSA | MDRS-2 total score* | / | / | / | / | / |
| Lopez et al. (2019) | UPDRS III | MMSE > 24;  *N* = 19 | Revised Observed Tasks of Daily Living (OTLD-R; finances) | / | MDRS-2 initiation* | MDRS-2 attention* | MDRS-2 memory* | n.s. | n.s. |
|  |  |  | OTLD-R (medication) | / | n.s. | n.s. | n.s. | n.s. | n.s. |
|  |  |  | OTLD-R (telephone) | / | n.s. | n.s. | n.s. | n.s. | n.s. |
| Manning et al. (2012) | NA | MoCA ≥ 25;  *N* = 26 | Hopkins Medication Schedule (HMS; total schedule) | n.s. | TMT-B & WCST-64 (perseverative errors & categories) | Digit Span Backward (WMS-III) | HVLT-R (learning & recall) | / | / |
|  |  |  | HMS (pillbox component) | n.s. | TMT-B & WCST-64 (perseverative errors & categories) | TMT-A | n.s. | / | / |
| De Oliveira et al. (2020) | * Age, education, & UPDRS III | Mixed (suspected mild PDD);  *N* = 32 | Direct Assessment of Functional Ability (DAFA) | MMSE &  MDRS total score* | MDRS Initiation | MDRS Attention | MDRS Memory | MDRS Construction | MDRS Concept-ualization |
| Pirogovsky et al. (2012) | NA | Mixed;  *N* = 33 | Medication Management Ability Assessment (MMAA) | MDRS total score | / | / | / | MIST | / |
|  |  |  | Advanced Finances Test (AFT) | MDRS total score | / | / | / | MIST | / |
| Pirogovsky et al. (2014) | NA | PD-MCI;  *N* = 41 | MMAA | n.s. | n.s. | n.s. | n.s. | n.s. | n.s. |
|  |  |  | UPSA (finances) | n.s. | n.s. | n.s. | n.s. | n.s. | n.s. |
| Schmitter-Edgecombe et al. (2022) | NA | PD-MCI;  *N* = 18 | Six Activities Task (total score) | / | Executive domain score | n.s. | n.s. | / | / |
|  |  |  | Six Activities Task (time) | / | Executive domain score | Attention domain score | n.s. | / | / |
| Schmitter-Edgecombe et al. (2024) | * Timed Up and Go Test & other cognitive domain scores | Non-PDD;  *N* = 38;  *N* = 37* | Day Out Task (time) | / | Executive domain score | / | n.s. | n.s. | n.s. |
|  |  |  | Day Out Task (performance) | / | Executive domain score* | / | Memory domain score | Visuo-cognitive domain score | Language domain score |
|  |  |  | Day Out Task (sequencing) | / | n.s. | / | Memory domain score | n.s. | n.s. |
| Sulzer et al. (2020) | NA | Non-PDD;  *N* = 35 | Erlangen Test of ADL in Mild Dementia and Mild Cognitive Impairment (ETAM; total score) | Total domain score & MoCA | Executive domain score | Attention domain score | Memory domain score | Visuo-cognitive domain score | n.s. |
|  |  |  | ETAM (medication) | Total domain score & MoCA | Executive domain score | Attention domain score | Memory domain score | n.s. | n.s. |
|  |  |  | ETAM (finances) | Total domain score & MoCA | n.s. | Attention domain score | Memory domain score | Visuo-cognitive domain score | n.s. |
| Sumida et al. (2021) | NA | PD-MCI;  *N* = 45; *N* = 44 (executive function, attention/working memory) | MMAA Original | / | n.s. | n.s. | Delayed memory domain score | n.s. | n.s. |
|  |  |  | MMAA Overall Errors | / | n.s. | n.s. | Delayed memory domain score | n.s. | n.s. |

Legend: CANTAB. Cambridge Neuropsychological Test Automated Battery, CERAD. Consortium to Establish a Registry for Alzheimer's Disease, EVOCAT. Categorical Evocation Subtest of the Barcelona Test, GDS. Geriatric Depression Scale, HVLT-R. Hopkins Verbal Learning Test-Revised, LEDD. Levodopa Equivalent Daily Dose, MDRS(-2). Mattis Dementia Rating Scale (2), MIST. Memory for Intentions Screening Test, Mixed. sample includes Non-PDD and potentially PDD, MMSE. Mini Mental State Exam, MoCA. Montreal Cognitive Assessment, NA. Not available, NAI. Nürnberger Altersinventar, Non-PDD. dementia was exclusion criterion, n.s. not significant, PDD. Parkinson’s disease dementia, PD-MCI. Parkinson’s disease with mild cognitive impairment, TAP. Testbatterie zur Aufmerksamkeitsprüfung, TMT. Trail Making Test, UPDRS III. Unified Parkinson’s Disease Rating Scale part III, VOSP. Visual Object and Space Perception Battery, WCST-64. Wisconsin Card Sorting Test-64 Card Version, WMS-III/-R. Wechsler Memory Scale-Third Edition/ -Revised, /. not assessed, *. corrected for covariates listed in covariates column

**Supplementary Table 3**. Selected (quasi-) randomized controlled trials (RCT) with a passive control group (CG) and (instrumental) activity of daily living (IADL) function as primary, secondary, or explorative outcome in Parkinson’s disease (PD)

| **Author** | **Design** | **Intervention** | **Age in years^a^** | **Disease duration (DD) in years**  **and H&Y^a^** | **Treatment duration & intensity^b^** | **ADL outcome** | **Treatment effects on ADL** | **Comments** |
| --- | --- | --- | --- | --- | --- | --- | --- | --- |
| **Physical intervention** | | | | | | | | |
| Conradsson et al. (2015) | Non-blind RCT | EG: HiBalance Program (n=51, DO=4) vs. CG: No intervention (n=49, DO=5) | EG: 72.9±6.0 CG: 73.6±5.3 | DD EG: NA CG: NA H&Y (%)  Stage 2/3: EG: 42.6/57.4 CG: 43.2/56.8 | EG: 60 min, 3 times per week for 10 weeks (30 sessions) | UPDRS-II | ↑ EG vs. CG (Pre vs. Post) | Dementia (MMSE<24) excluded, ability to walk without assistance |
| Choi (2016) | Single-blind RCT | EG: Tai Chi (n=11, DO=0) vs. CG: Waiting list (n=9, DO=0) | EG: 60.8±7.6  CG: 65.5±6.8 | DD  EG: 5.2±2.7  CG: 5.2±2.7  H&Y  EG: 1.6±0.6  CG: 1.8±0.3 | EG: Tai Chi - 60 min, 2 times per week for 12 weeks (24 sessions); Home-based activity – 60 min, 1 time per week for 12 weeks (12 sessions) | Unified Parkinson’s Disease Rating Scale part III (UPDRS-III) | ↑ EG (Pre vs. Post) |  |
| da Silva et al. (2023) | Single-blind RCT | EG: Aquatic dual task training (n=14, DO=0) vs. CG: No intervention (n=11, DO=0) | EG: 63±13 CG: 64±13 | DD EG: 6.4±3.8 CG: 5.8±3.5 H&Y  EG: 3±1 CG: 3±1 | EG: 40 min, 2 times per week for 10 weeks (20 sessions) | UPDRS-II (primary outcome) | ↑ EG vs. CG (Pre vs. Post) | Ability to follow instruction (MMSE), no wheelchair; stable levodopa medication during trial |
| Duncan and Earhart (2012) | Single-blind RCT | EG: Tango dance therapy (n=32, DO=6-16; community-based classes) vs. CG: No intervention (n=30, DO=4-11) | EG: 69.3±1.9  CG: 69.0±1.5 | DD  EG: 5.8±1.1  CG: 7.0±1.0  H&Y  EG: 2.6±0.1  CG: 2.5±0.1 | EG: 60 min, 2 times per week for 12 months | UPDRS-II | No effect |  |
| Ellis et al. (2005) | Single-blind cross-over RCT | EG: Physical therapy & standard medication treatment (n=33, DO=unclear) vs. CG: Standard medication treatment (n=32, DO=unclear) | EG: 63±8.8 CG: 64±8.4 | DD EG: NA CG: NA H&Y  EG: 2.4±0.5 CG: 2.5±0.5 | EG: 90 min, 2 times per week for 6 weeks (12 sessions) | UPDRS-II | ↑ EG vs. CG (Pre vs. Post vs. Follow-Up) | Dementia (MMSE<24) excluded, ability to walk without assistance, no participation in any other physical intervention, stable medication during trial |
| Foster et al. (2013) | Single-blind RCT | EG: Tango dance therapy (n=26, DO=10) vs. CG: No intervention (n=26, DO=7) | EG: 69.3±9.4 CG: 69.0±7.8 | DD  EG: 5.8±5.4 CG: 7.0±4.8 H&Y (%)  Stage 2/2.5/3/4:  EG: 42.3/38.5/19.2/0.0 CG: 34.7/42.3/19.2/3.8 | EG: 60 min, 2 times per week for 12 months | Activity Card Sort Test (ACS; primary outcome) | ↑ EG & CG (Pre vs. Post); ↑ EG (Pre vs. Post) in ACS social activities | Psychiatric disorders excluded, ability to walk without assistance for 3 m |
| Nieuwboer et al. (2007) | Single-blind cross-over RCT | EG: Home-based gait cueing training (n=77, DO=1) vs. CG: Waiting list (n=77, DO=0) | Median, IQR  EG: 67.5, 61.5-72 CG: 69, 62.5-73 | DD (Median, IQR) EG: 7, 4-11 CG: 8, 4-12 H&Y Stage 2/3/4:  EG: 51.3/38.2/10.5  CG: 41.6/45.5/13.0 | EG: 30 min, ~3 times per week for 3 weeks (9 sessions) | Nottingham Extended ADL Scale (NEADL) | No effect | Dementia (MMSE<24) excluded, ability to walk without assistance |

**[Continued] Supplementary Table 3**. Selected (quasi-) randomized controlled trials (RCT) with a passive control group (CG) and (instrumental) activity of daily living (IADL) function as primary, secondary, or explorative outcome in Parkinson’s disease (PD)

| **Author** | **Design** | **Intervention** | **Age in years^a^** | **Disease duration (DD) in years**  **and H&Y^a^** | **Treatment duration & intensity^b^** | **ADL outcome** | **Treatment effects on ADL** | **Comments** |
| --- | --- | --- | --- | --- | --- | --- | --- | --- |
| Nocera et al. (2013) | Single-blind RCT | EG: Tai Chi (n=17, DO=2) vs. CG: No intervention (n=6, DO=0) | EG: 66±11 CG: 65±7 | DD EG: 8.1±5.4 CG: 6.8±1.8 H&Y  All participants: Stage 2-3 | EG: 60 min, 3 times per week for 16 weeks (48 sessions) | ADL part of Parkinson’s Disease Questionnaire (PDQ-39) | ↑ EG (change scores, Pre vs. Post) | Dementia (MMSE<26) and depression (BDI-II≥17) excluded, ability to walk without assistance, no participation in any other physical intervention |
| **Cognitive training** | | | | | | | | |
| Folkerts et al. (2018) | Cross-over RCT, accessor blinded for Post-test | EG: Cognitive stimulation (n=6, DO=1) vs. CG: Routine care (n=6, DO=1) | EG: 76.7±5.5 CG: 76.5±8.9 | DD EG: 6.0±3.2 CG: 6.2±4.1 H&Y (%) Stage: 2-2.5/3/4/5  EG: 33.4/0/33.3/33.3  CG: 16.7/33.3/33.3/16.7 | EG: 60 min, 2 times per week for 8 weeks (16 sessions) | Barthel Index (BI) | ↓ EG & CG (Pre vs. Post vs. Follow-Up) | Only participants with PDD (MDS criteria); depression (GDS-15≥5) excluded |
| Lawrence et al. (2018) | Non-blinded RCT | EG1: Standard cognitive training (n=7, DO=0-2) vs. EG2: Tailored cognitive training (n=7, DO=0-1) vs. EG3: tDCS (n=7, DO=0) vs. EG4: Standard cognitive training + tDCS, (n=7, DO=0) vs. EG5: Tailored cognitive training + tDCS (n=7, DO=0) vs. CG: Passive CG (n=7, DO=0-1) | EG1: 68.1±8.7  EG2: 66.6±5.2  EG3: 72.0±6.5  EG4: 63.6±15.7  EG5: 67.4±6.4  CG: 72.3±6.2 | DD:  EG1: 5.3±4.2 EG2: 5.8±5.0  EG3: 5.5±5.7  EG4: 6.8±4.6  EG5: 4.4±2.7  CG: 5.4±4.1  HY:  NA | Cognitive training: computer-based training for 45 minutes,  3 times per week for 4 weeks (12 sessions); tDCS: 20 minutes of stimulation, 1 time per week for 4 weeks (4 sessions) | UPDRS-II | ↑ EG1 vs. CG (Pre vs. Post); ↑ EG4 vs. CG (Pre vs. Post) | Only participants with PD-MCI (  (MDS criteria) and cognitive deficits that did not interfere with functional independence (UPDRS-II<3) |
| **Multi-domain intervention** | | | | | | | | |
| Frazzitta et al. (2012) | Single-blind RCT | EG: “multidisciplinary  intensive rehabilitation treatment” (MIRT)=training involving muscle stretching, balance exercises, treadmill, cross-trainer, ergometer, & occupational ADL therapy (n=25, DO=0) vs. CG: Routine care (n=25, DO=0) | EG: 72±7 CG: 70±7 | DD EG: 8±3  CG: 9±3 H&Y  All participants: Stage 3 | EG: 60 min, 3 times per day for 5 days per week for 4 weeks (60 sessions) | UPDRS-II (primary outcome) | ↑ EG vs. CG (Pre vs. Post vs. Follow-Up) | Dementia (MMSE≤26) excluded, ability to walk without assistance |
| Frazzitta et al. (2015) | Single-blind RCT | EG: MIRT (n=20, DO=4) vs. CG: Routine care (n=15, DO=5) | EG: 69±6 CG: 68±8 | DD  De novo PD diagnosis H&Y  EG: 1.2±0.3 CG: 1.1±0.3 | EG: 60 min, 3 times per day for 5 days per week for 4 weeks (60 sessions) | UPDRS-II (primary outcome) | ↑ EG vs. CG (Pre vs. Post vs. Follow-Up 1 vs. Follow-Up 2 vs. Follow-Up 3) | Only participants with de novo PD diagnosis; dementia (MMSE≤26) excluded |

**[Continued] Supplementary Table 3**. Selected (quasi-) randomized controlled trials (RCT) with a passive control group (CG) and (instrumental) activity of daily living (IADL) function as primary, secondary, or explorative outcome in Parkinson’s disease (PD)

| **Author** | **Design** | **Intervention** | **Age in years^a^** | **Disease duration (DD) in years**  **and H&Y^a^** | **Treatment duration & intensity^b^** | **ADL outcome** | **Treatment effects on ADL** | **Comments** |
| --- | --- | --- | --- | --- | --- | --- | --- | --- |
| Sturkenboom et al. (2013) | RCT, assessor blinded for Post-test | EG: Individualized home-based occupational therapy based on patients’ and caregivers’ priorities (n=29 patients, DO=2; n=29; caregivers, DO=3) vs. CG: Waiting list (n=14 patients, DO=1; n=14 caregivers, DO=1) | EG: 66.7±11.8  CG: 68.5±9.6 | DD  EG: 7.5±7.1  CG: 6.9±4.3  H&Y (%)  Stage: 1/2/3/4/5  EG: 14.8/55.6/25.9/0.0/3.7  CG: 0.0/84.6/15.4/0.0/0.0 | EG: 45-60 min, variable number of times per week, depending on the priorities, for 10 weeks (max. 16 sessions) | Canadian Occupational Performance Measure (COPM; primary outcome), Assessment of Motor and Process Scale process skills (AMPS; primary outcome) | No effect in COPM; no effect in AMPS process skills | Only participants with ADL difficulties (regarding self-care, domestic activities, work, or leisure); no participation in any other occupational rehabilitation within the past 12 months, no participation in any other intervention, unblinding for 33% of participants |
| Sturkenboom et al. (2014) | Single-blind RCT, stratified by various aspects (e.g. region, age, gender, ADL performance) | EG: Individualized home-based occupational therapy (n=124 patients, DO=3-6; n=117 caregivers, DO=3-4) vs. CG: Routine care (n=67 patients, DO=10-16; n=63 caregivers, DO=3-10) | Median, IQR  EG: 71.0, 63.3-76.0 CG: 70.0, 63.3-75.0 | DD (Median, IQR) EG: 6.0, 4.0-10.0 CG: 6.0, 3.0-11.0 H&Y (%)  Stage: 1/2/3/4/5 EG: 25/37/35/2/1 CG: 22/48/24/6/0 | EG: 60 min, variable number of times per week for 10 weeks (max. 16 sessions) | COPM (primary outcome), ACS, Perceive Recall Plan Perform System phase 1 (PRPP-1) | ↑ EG vs. CG (Pre vs. Post, Pre vs. Follow-Up) in COPM; ↑ EG vs. CG (Pre vs. Post) in instrumental ADL of ACS; no effect in PRPP-1 | Only participants with difficulties in meaningful daily activities; dementia (MMSE<24) excluded; no participation in any other occupational rehabilitation within the past 3 months |
| **ADL-related intervention** | | | | | | | | |
| Daley et al. (2014) | RCT, blinding of statistician, stratification by availability of caregiver | EG: Patient-caregiver medication adherence therapy (n=38 patients, DO=0; n=25 caregivers, DO=1) vs. CG: Routine care (n=38 patients, DO=0; n=23 caregivers, DO=1) | EG: 72.2±9.5 CG: 71.6±8.3 | DD EG: 8.7±6.4 CG: 7.8±4.2  H&Y  EG: 2.1±1.1 CG: 2.2±1.1 | EG: 30-60 min, 1 time per week for 7 weeks (7 sessions) | ADL part of PDQ-39, Morisky Medication Adherence Scale (MMAS-4; primary outcome) & UPDRS-II | ↑ EG vs. CG (Pre vs. Post) in ADL part of PDQ-39; ↑ EG vs. CG (Pre vs. Post) in MMAS-4; ↑ EG vs. CG (Pre vs. Post) in UPDRS-II | Only participants with MMAS-4≥1; dementia excluded; stable medication during trial |

Legend: ^a^. If not otherwise indicated, values given as Mean±SD, ^b^. If not otherwise indicated, information applies to both EG and CG, BDI-II. Beck Depression Inventory II, CG. Control group, DD. Disease duration, DO. Drop-out, EG. Experimental (treatment) group, GDS. Geriatric Depression Scale, H&Y. Hoehn & Yahr stage, m. Meter, min. Minute, NA. Not available, MDS. Movement Disorder Society, MMSE. Mini Mental State Exam, PDD. Parkinson’s disease dementia, tDCS. transcranial direct current stimulation.

**References**

Becker S, Maarouf N, Kibreab M, Hammer T, Kathol I, Monchi O, Callahan BL (2023) 66 Association of Executive Functions and Instrumental Activities of Daily Living in Parkinson’s Disease. Journal of the International Neuropsychological Society 29 (s1):576-577. doi:https://doi.org/10.1017/s1355617723007373Choi HJ (2016) Effects of therapeutic Tai chi on functional fitness and activities of daily living in patients with Parkinson disease. J Exerc Rehabil 12 (5):499-503. doi:https://doi.org/10.12965/jer.1632654.327

Choi SM, Yoon GJ, Jung HJ, Kim BC (2019) Analysis of characteristics affecting instrumental activities of daily living in Parkinson's disease patients without dementia. Neurol Sci 40 (7):1403-1408. doi:https://doi.org/10.1007/s10072-019-03860-0

Cholerton B, Poston KL, Tian L, Quinn JF, Chung KA, Hiller AL, Hu SC, Specketer K, Montine TJ, Edwards KL, Zabetian CP (2020) Participant and Study Partner Reported Impact of Cognition on Functional Activities in Parkinson's Disease. Mov Disord Clin Pract 7 (1):61-69. doi:https://doi.org/10.1002/mdc3.12870

Conradsson D, Lofgren N, Nero H, Hagstromer M, Stahle A, Lokk J, Franzen E (2015) The Effects of Highly Challenging Balance Training in Elderly With Parkinson's Disease: A Randomized Controlled Trial. Neurorehabil Neural Repair 29 (9):827-836. doi:https://doi.org/10.1177/1545968314567150

da Silva AZ, Iucksch DD, Israel VL (2023) Aquatic Dual-Task Training and Its Relation to Motor Functions, Activities of Daily Living, and Quality of Life of Individuals With Parkinson's Disease: A Randomized Clinical Trial. Health Serv Insights 16:11786329231180768. doi:https://doi.org/10.1177/11786329231180768

Daley DJ, Deane KH, Gray RJ, Clark AB, Pfeil M, Sabanathan K, Worth PF, Myint PK (2014) Adherence therapy improves medication adherence and quality of life in people with Parkinson's disease: a randomised controlled trial. Int J Clin Pract 68 (8):963-971. doi:https://doi.org/10.1111/ijcp.12439

de Oliveira GSR, Bressan L, Balarini F, Jesuino ESRS, Brito M, Foss MP, Santos-Lobato BL, Tumas V (2020) Direct and indirect assessment of functional abilities in patients with Parkinson's disease transitioning to dementia. Dement Neuropsychol 14 (2):171-177. doi:https://doi.org/10.1590/1980-57642020dn14-020011

Duncan RP, Earhart GM (2012) Randomized controlled trial of community-based dancing to modify disease progression in Parkinson disease. Neurorehabil Neural Repair 26 (2):132-143. doi:https://doi.org/10.1177/1545968311421614

Ellis T, de Goede CJ, Feldman RG, Wolters EC, Kwakkel G, Wagenaar RC (2005) Efficacy of a physical therapy program in patients with Parkinson's disease: a randomized controlled trial. Arch Phys Med Rehabil 86 (4):626-632. doi:https://doi.org/10.1016/j.apmr.2004.08.008

Fellows RP, Schmitter-Edgecombe M (2019) Multimethod assessment of everyday functioning and memory abilities in Parkinson's disease. Neuropsychology 33 (2):169-177. doi:https://doi.org/10.1037/neu0000505

Fernández-Baizán C, García-Nevares A, Díaz-Cáceres E, Menéndez-González M, Arias JL, Méndez M (2022) Activities of Daily Living in Parkinson Disease: Are They Related to Short Term and Working Memory and Visuospatial Abilities? Topics in Geriatric Rehabilitation 38 (4):285-295. doi:https://doi.org/10.1097/TGR.0000000000000376

Folkerts AK, Dorn ME, Roheger M, Maassen M, Koerts J, Tucha O, Altgassen M, Sack AT, Smit D, Haarmann L, Kalbe E (2018) Cognitive Stimulation for Individuals with Parkinson's Disease Dementia Living in Long-Term Care: Preliminary Data from a Randomized Crossover Pilot Study. Parkinsons Dis 2018:8104673. doi:https://doi.org/10.1155/2018/8104673

Foster ER (2014) Instrumental activities of daily living performance among people with Parkinson's disease without dementia. Am J Occup Ther 68 (3):353-362. doi:https://doi.org/10.5014/ajot.2014.010330

Foster ER, Doty T (2021) Cognitive Correlates of Instrumental Activities of Daily Living Performance in Parkinson Disease Without Dementia. Arch Rehabil Res Clin Transl 3 (3):100138. doi:https://doi.org/10.1016/j.arrct.2021.100138

Foster ER, Golden L, Duncan RP, Earhart GM (2013) Community-Based Argentine Tango Dance Program Is Associated With Increased Activity Participation Among Individuals With Parkinson's Disease. Archives of Physical Medicine and Rehabilitation 94 (2):240-249. doi:https://doi.org/10.1016/j.apmr.2012.07.028

Frazzitta G, Bertotti G, Riboldazzi G, Turla M, Uccellini D, Boveri N, Guaglio G, Perini M, Comi C, Balbi P, Maestri R (2012) Effectiveness of intensive inpatient rehabilitation treatment on disease progression in parkinsonian patients: a randomized controlled trial with 1-year follow-up. Neurorehabil Neural Repair 26 (2):144-150. doi:https://doi.org/10.1177/1545968311416990

Frazzitta G, Maestri R, Bertotti G, Riboldazzi G, Boveri N, Perini M, Uccellini D, Turla M, Comi C, Pezzoli G, Ghilardi MF (2015) Intensive rehabilitation treatment in early Parkinson's disease: a randomized pilot study with a 2-year follow-up. Neurorehabil Neural Repair 29 (2):123-131. doi:https://doi.org/10.1177/1545968314542981

Giovannetti T, Britnell P, Brennan L, Siderowf A, Grossman M, Libon DJ, Bettcher BM, Rouzard F, Eppig J, Seidel GA (2012) Everyday action impairment in Parkinson's disease dementia. J Int Neuropsychol Soc 18 (5):787-798. doi:https://doi.org/10.1017/S135561771200046X

Glonnegger H, Beyle A, Cerff B, Graber S, Csoti I, Berg D, Liepelt-Scarfone I (2016) The Multiple Object Test as a Performance Based Tool to Assess Cognitive Driven Activity of Daily Living Function in Parkinson's Disease. J Alzheimers Dis 53 (4):1475-1484. doi:https://doi.org/10.3233/JAD-160173

Higginson CI, Lanni K, Sigvardt KA, Disbrow EA (2013) The contribution of trail making to the prediction of performance-based instrumental activities of daily living in Parkinson's disease without dementia. J Clin Exp Neuropsychol 35 (5):530-539. doi:https://doi.org/10.1080/13803395.2013.798397

Holden SK, Medina LD, Hoyt B, Sillau SH, Berman BD, Goldman JG, Weintraub D, Kluger BM (2018) Validation of a performance-based assessment of cognitive functional ability in Parkinson's disease. Mov Disord 33 (11):1760-1768. doi:https://doi.org/10.1002/mds.27487

Lawrence BJ, Gasson N, Johnson AR, Booth L, Loftus AM (2018) Cognitive Training and Transcranial Direct Current Stimulation for Mild Cognitive Impairment in Parkinson's Disease: A Randomized Controlled Trial. Parkinsons Dis 2018:4318475. doi:https://doi.org/10.1155/2018/4318475

Lopez FV, Ferencz B, Rohl BY, Trifilio E, Scott B, Okun MS, Marsiske M, Bowers D (2019) Everyday functioning in Parkinson's disease: Evidence from the Revised-Observed Tasks of Daily Living (OTDL-R). Parkinsonism Relat Disord 60:167-170. doi:https://doi.org/10.1016/j.parkreldis.2018.08.026

Manning KJ, Clarke C, Lorry A, Weintraub D, Wilkinson JR, Duda JE, Moberg PJ (2012) Medication management and neuropsychological performance in Parkinson's disease. Clin Neuropsychol 26 (1):45-58. doi:https://doi.org/10.1080/13854046.2011.639312

Nieuwboer A, Kwakkel G, Rochester L, Jones D, van Wegen E, Willems AM, Chavret F, Hetherington V, Baker K, Lim I (2007) Cueing training in the home improves gait-related mobility in Parkinson's disease: the RESCUE trial. Journal of Neurology, Neurosurgery & Psychiatry 78 (2):134-140. doi:https://doi.org/10.1136/jnnp.200X.097923

Nocera JR, Amano S, Vallabhajosula S, Hass CJ (2013) Tai Chi Exercise to Improve Non-Motor Symptoms of Parkinson's Disease. J Yoga Phys Ther 3. doi:https://doi.org/10.4172/2157-7595.1000137

Pirogovsky E, Schiehser DM, Obtera KM, Burke MM, Lessig SL, Song DD, Litvan I, Filoteo JV (2014) Instrumental activities of daily living are impaired in Parkinson's disease patients with mild cognitive impairment. Neuropsychology 28 (2):229-237. doi:https://doi.org/10.1037/neu0000045

Pirogovsky E, Woods SP, Vincent Filoteo J, Gilbert PE (2012) Prospective memory deficits are associated with poorer everyday functioning in Parkinson's disease. J Int Neuropsychol Soc 18 (6):986-995. doi:https://doi.org/10.1017/S1355617712000781

Puente AN, Cohen ML, Aita S, Brandt J (2016) Behavioral Ratings of Executive Functioning Explain Instrumental Activities of Daily Living beyond Test Scores in Parkinson's Disease. Clin Neuropsychol 30 (1):95-106. doi:https://doi.org/10.1080/13854046.2015.1133847

Schmitter-Edgecombe M, Brown K, Chilton RC, Whiteley N, Greeley D (2024) Naturalistic assessment of everyday multitasking in Parkinson’s disease with and without mild cognitive impairment. The Clinical Neuropsychologist:1-21. doi:https://doi.org/10.1080/13854046.2024.2325681

Schmitter-Edgecombe M, McAlister C, Greeley D (2022) A Comparison of Functional Abilities in Individuals with Mild Cognitive Impairment and Parkinson's Disease with Mild Cognitive Impairment Using Multiple Assessment Methods. J Int Neuropsychol Soc 28 (8):798-809. doi:https://doi.org/10.1017/S1355617721001077

Sturkenboom IH, Graff MJ, Borm GF, Veenhuizen Y, Bloem BR, Munneke M, Nijhuis-van der Sanden MW (2013) The impact of occupational therapy in Parkinson's disease: a randomized controlled feasibility study. Clin Rehabil 27 (2):99-112. doi:https://doi.org/10.1177/0269215512448382

Sturkenboom IH, Graff MJ, Hendriks JC, Veenhuizen Y, Munneke M, Bloem BR, Nijhuis-van der Sanden MW, group OTs (2014) Efficacy of occupational therapy for patients with Parkinson's disease: a randomised controlled trial. Lancet Neurol 13 (6):557-566. doi:https://doi.org/10.1016/S1474-4422(14)70055-9

Sulzer P, Baumer A, Hoang HG, Becker S, Lonneker HD, Graessel E, Liepelt-Scarfone I (2020) Assessment of Cognitive-Driven Performance-Based Activities of Daily Living Dysfunction in Parkinson's Disease. J Int Neuropsychol Soc 26 (4):430-440. doi:https://doi.org/10.1017/S1355617719001322

Sumida CA, Lopez FV, Van Etten EJ, Whiteley N, Moore RC, Litvan I, Lessig S, Gilbert PE, Schmitter-Edgecombe M, Filoteo JV, Schiehser DM (2021) Medication Management Performance in Parkinson's Disease: Examination of Process Errors. Arch Clin Neuropsychol 36 (7):1307-1315. doi:https://doi.org/10.1093/arclin/acab004
